# Fine-grained Chinese dish recognition on CNFOOD-241: A discriminative learning approach for improving category-level discrimination

**DOI:** 10.1371/journal.pone.0358248

**Published:** 2026-09-25

**Authors:** Xin Xiong, Jinqiang Liao, Xiao Mo, JianFeng He

**Affiliations:** Faculty of Information Engineering and Automation, Kunming University of Science and Technology, Kunming, China; The University of Lahore, PAKISTAN

## Abstract

Accurate recognition of food images is essential for image-based dietary assessment, nutritional tracking, and digital health applications. However, closed-set Chinese dish recognition remains challenging because many categories in the CNFOOD-241 dataset exhibit strong visual similarity, substantial intra-class variation, and subtle inter-class differences. In this study, we present an integrated discriminative training strategy for fine-grained Chinese dish recognition using RegNetY-32GF as the student backbone. Rather than proposing a new backbone architecture, the study focuses on improving category-level discrimination under a fixed convolutional architecture by combining teacher-guided knowledge distillation, margin-based classification, triplet-based embedding regularization, and mixed-sample augmentation. Across five random seeds, the proposed full model achieved 84.37 + /- 0.29% Top-1 accuracy, 97.66 + /- 0.11% Top-5 accuracy, 83.41 + /- 0.57% macro-F1, and 84.27 + /- 0.30% weighted-F1. Paired bootstrap analysis showed a positive Top-1 improvement of 0.651 percentage points over the baseline, with a 95% confidence interval of [0.491, 0.817], and the mean class-wise Delta F1 across five seeds was significantly greater than zero according to the Wilcoxon signed-rank test (p = 0.017803). These findings suggest that the proposed framework provides a modest improvement in category-level discrimination on CNFOOD-241, with statistically supported evidence at the dataset level, although the magnitude of improvement varies across individual categories. The training code, evaluation scripts, derived result files, and trained model weights are publicly available at the project repository and GitHub Release.

## Introduction

### Background

Food image recognition has become an increasingly important component of image-based dietary assessment, digital nutrition tools, and intelligent lifestyle monitoring. In health-related scenarios, automatic recognition of meals from smartphone images can support food logging, calorie estimation, and long-term nutrition management. These applications are particularly relevant for populations requiring structured dietary monitoring, including individuals with obesity, diabetes, and other chronic metabolic disorders [1–4].

Compared with general food recognition, Chinese dish recognition is especially challenging because real-world dishes vary substantially in cooking style, plating, illumination, viewpoint, and ingredient visibility. At the same time, many dish categories differ only in subtle ingredient combinations or local appearance cues. As a result, a model that performs well on coarse food classification may still struggle when asked to distinguish visually similar dish categories [5–7].

### Related work on CNFOOD-241 and fine-grained food recognition

CNFOOD-241 is a large-scale Chinese dish recognition dataset containing 241 categories and more than 190,000 images. The original benchmark reported strong performance for several deep convolutional models, with ResNeXt101_32x32d achieving 82.05% Top-1 accuracy and 97.13% Top-5 accuracy, while a stacking-based fusion strategy reached 82.88% Top-1 accuracy. More recent studies have continued to use CNFOOD-241 as a benchmark for fine-grained Chinese dish recognition and related downstream modeling; for example, Feng et al. reported a RegNetY-32GF-based pipeline for classification and nutrient estimation, whereas ResVMamba further emphasized the fine-grained nature of the dataset and its complex category-level differences [8–11].

Although these studies demonstrated that high-capacity architectures can achieve competitive overall recognition performance, overall accuracy alone does not fully describe behavior on genuinely difficult fine-grained categories. In a dataset such as CNFOOD-241, a modest gain in Top-1 accuracy may still hide substantial differences in how well a method separates visually similar dish pairs. This concern becomes even more important when category frequencies are imbalanced, because aggregate accuracy may obscure instability in difficult or under-represented classes [12–14]. Therefore, evaluation of Chinese dish recognition methods should consider not only overall accuracy but also whether category-level discrimination is improved under fine-grained conditions.

### Motivation and objective of this study

The objective of this study was not to introduce a completely new backbone architecture, but to improve category-level discrimination on CNFOOD-241 through a more targeted training strategy built on a strong convolutional baseline. We hypothesized that, under a fixed backbone architecture, encouraging more discriminative feature learning and more stable class separation would improve recognition performance on genuinely fine-grained dish categories [5,15–17].

To test this hypothesis, we adopted RegNetY-32GF as the baseline backbone and designed an integrated discriminative training strategy that combines margin-based classification, teacher-guided knowledge distillation, embedding regularization, and mixed-sample augmentation. The contributions of this study are threefold. First, we established a strong RegNetY-32GF baseline for closed-set Chinese dish recognition on CNFOOD-241 and evaluated the proposed method with repeated experiments over five random seeds. Second, we examined the contribution of the discriminative components through single-component, pairwise, and multi-component ablation analyses, while avoiding an overstatement of uniform synergy among modules. Third, we provided category-level analysis based on class-wise F1 scores, confusion-derived hard-category evidence, and statistically tested Delta F1 distributions to better characterize fine-grained recognition behavior beyond aggregate accuracy.

## Materials and Methods

### Problem definition

This study addressed the problem of closed-set Chinese dish recognition on the CNFOOD-241 dataset. Given an input food image I, the goal was to assign it to one of 241 predefined dish categories (C = 241). The task can be formulated as learning a mapping:


$$\begin{array}{c} \hat{y}=f(I),\hat{y}\in \text{1},\text{2},\ldots ,\text{2}\text{4}\text{1} \end{array}$$ (1)

where ŷ denotes the predicted category label. Because many dish categories differ only in subtle visual cues, the task is not only a standard image classification problem but also a fine-grained representation learning problem.

### Overall framework

**Fig 1:**
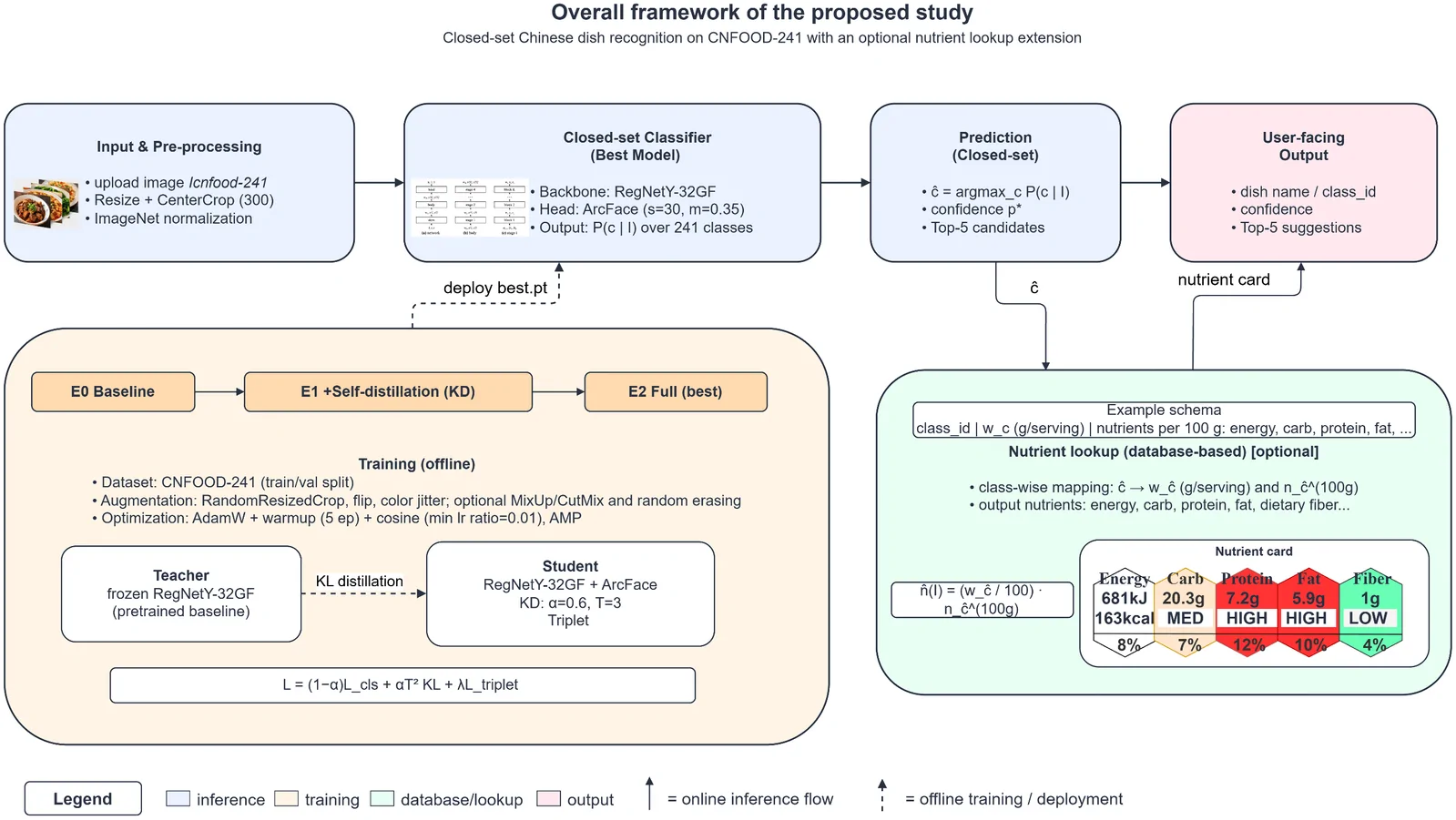
Overall framework of the proposed closed-set Chinese dish recognition pipeline, including a conceptual nutrient lookup extension that was not evaluated in this study. The schematic layout was created by the authors in draw.io, and the food photographs shown as visual examples were taken by the authors.

The overall study framework is illustrated in Fig 1. The pipeline included image input, closed-set Chinese dish recognition, category prediction, and a conceptual downstream nutrient lookup extension. This downstream extension was included to show a possible application pathway and was not experimentally evaluated as a core contribution of the present study.

The main contribution of this work lies in the recognition model and the integrated discriminative training strategy. The optional nutrient card module was retained only as a conceptual downstream extension rather than a core modeling contribution, and no quantitative claims are made regarding nutrient estimation in this study. In this extension, the recognized dish category can be linked to a predefined nutrition database entry to present approximate dish-level nutrient information. This design is conceptually consistent with image-based dietary assessment, but it requires separate validation before being used as a nutrition-estimation system.

### Dataset and preprocessing

Experiments were conducted on CNFOOD-241, a large-scale Chinese dish recognition dataset containing 191,811 images from 241 categories [8,9]. The dataset is publicly available through Mendeley Data (https://doi.org/10.17632/fspyss5zbb.1) and is distributed under the CC BY-NC 3.0 licence. Following the benchmark setting, the dataset was used for closed-set classification. The dataset is challenging because many classes show strong visual overlap, while samples from the same class can also vary substantially in appearance. Before training and inference, each image was resized to a fixed input resolution and normalized using ImageNet statistics. The original raw images were accessed from the dataset source and are not redistributed by this study; all derived outputs generated by our experiments are provided through the project repository.

### Dataset difficulty and fine-grained challenges

Although CNFOOD-241 is often used as a food-classification benchmark, its practical difficulty is strongly shaped by fine-grained inter-class similarity. Many categories share overlapping visual patterns in color, texture, garnish, plating style, or ingredient arrangement, whereas a single category may also exhibit large appearance variation due to cooking style, serving conditions, and image acquisition differences. Recent studies related to CNFOOD-241 have also emphasized its fine-grained and visually confusing nature [5,10,11].

**Fig 2:**
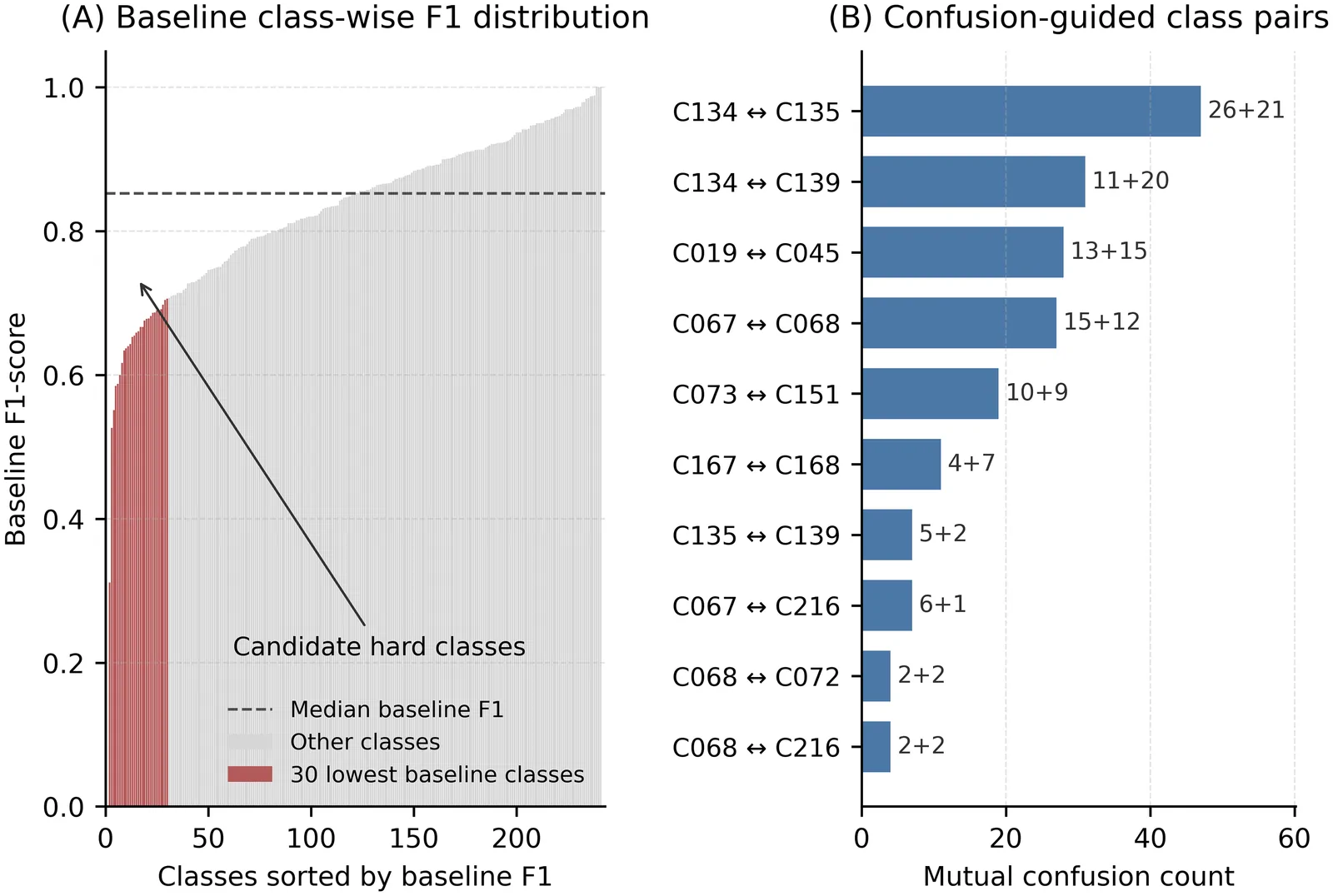
Prediction-based evidence of dataset difficulty and category-level confusion in CNFOOD-241.

Fig 2 provides prediction-based evidence of dataset difficulty and category-level confusion in CNFOOD-241. Rather than reproducing original dataset photographs, the figure summarizes class-wise baseline F1 scores and confusion-guided hard category pairs derived from the baseline prediction outputs. Candidate hard categories were first identified from the 30 lowest-performing baseline classes and then refined using mutual-confusion counts extracted from the confusion matrix. This result-based visualization illustrates why high overall accuracy does not necessarily imply strong category-level discrimination: some categories remain difficult because visually similar classes produce frequent cross-pair prediction errors.

Panel A shows the class-wise F1-score distribution of the baseline model across all 241 categories, with the 30 lowest-performing classes highlighted as candidate hard categories. Panel B shows confusion-guided class pairs involving these hard categories, extracted from baseline prediction errors. Mutual confusion was defined as the sum of cross-pair misclassifications between two categories. This result-based figure does not reproduce original CNFOOD-241 photographs.

These prediction-derived hard-category patterns motivated a stronger emphasis on category-level discrimination in both model design and evaluation.

### Baseline network

We adopted RegNetY-32GF as the baseline backbone. RegNet models are a family of convolutional networks derived from a regularized network design space, and torchvision provides an official implementation of regnet_y_32gf. The corresponding configuration can be summarized by depth = 20, ${\text{w}}_{\text{0}}$ = 232, ${\text{w}}_{\text{a}}$ = 115.89, ${\text{w}}_{\text{m}}$ = 2.53, group width = 232, and $\text{s}{\text{e}}_{\text{ra}tio}$ = 0.25, forming a four-stage architecture [15].

The baseline model consisted of the RegNetY-32GF backbone followed by a classification head for 241-way prediction. This model served as the reference point for evaluating the contribution of the proposed discriminative learning mechanisms. To provide a broader architectural context during revision, we additionally evaluated representative transformer-family baselines, including ViT-B/16 and Swin-B, under the same data split and input resolution. Other backbone-family examples frequently used in image classification include ResNet, DenseNet, ResNeXt, and EfficientNet [18–21].

### Main architecture of the student model

**Fig 3:**
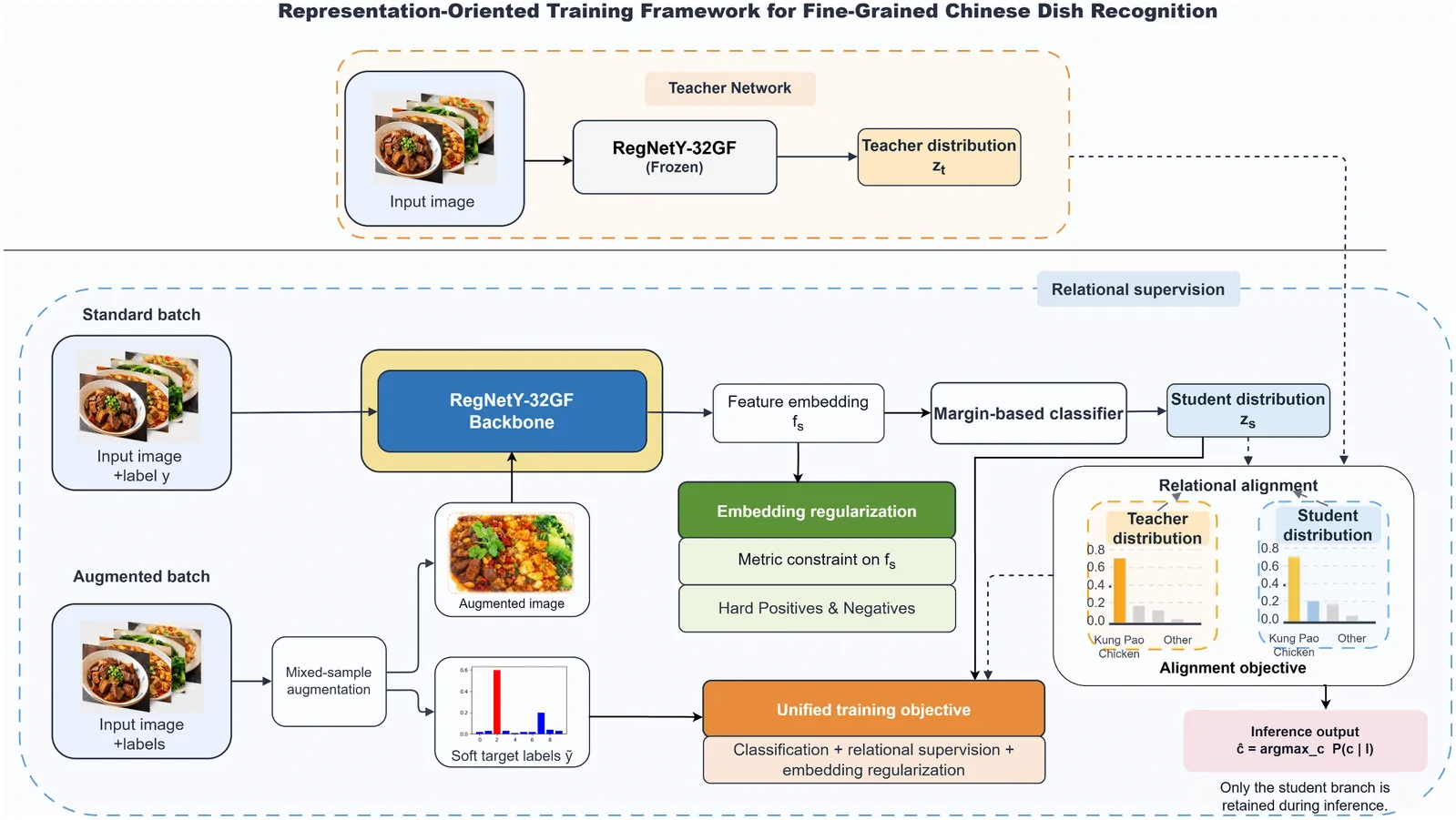
Backbone architecture of the student model. The schematic architecture was created by the authors in draw.io, and any food photographs shown as visual examples were taken by the authors.

The deployed student model architecture is shown in Fig 3.

The student model was built on the RegNetY-32GF backbone and comprised a stem convolution, four hierarchical feature extraction stages, global average pooling, a feature embedding layer, and a margin-based classification head. According to the RegNetY-32GF configuration, the stage widths were [232, 696, 1392, 3712] and the corresponding stage depths were [1,2,5,12], with group width fixed at 232. The pooled backbone representation was transformed into an embedding used for discriminative classification.

The explicit separation between the main learning model architecture and the training design was intended to distinguish the deployed inference model from the auxiliary mechanisms used only during optimization. During inference, only the trained student branch was retained.

### Coordinated discriminative training design

**Fig 4:**
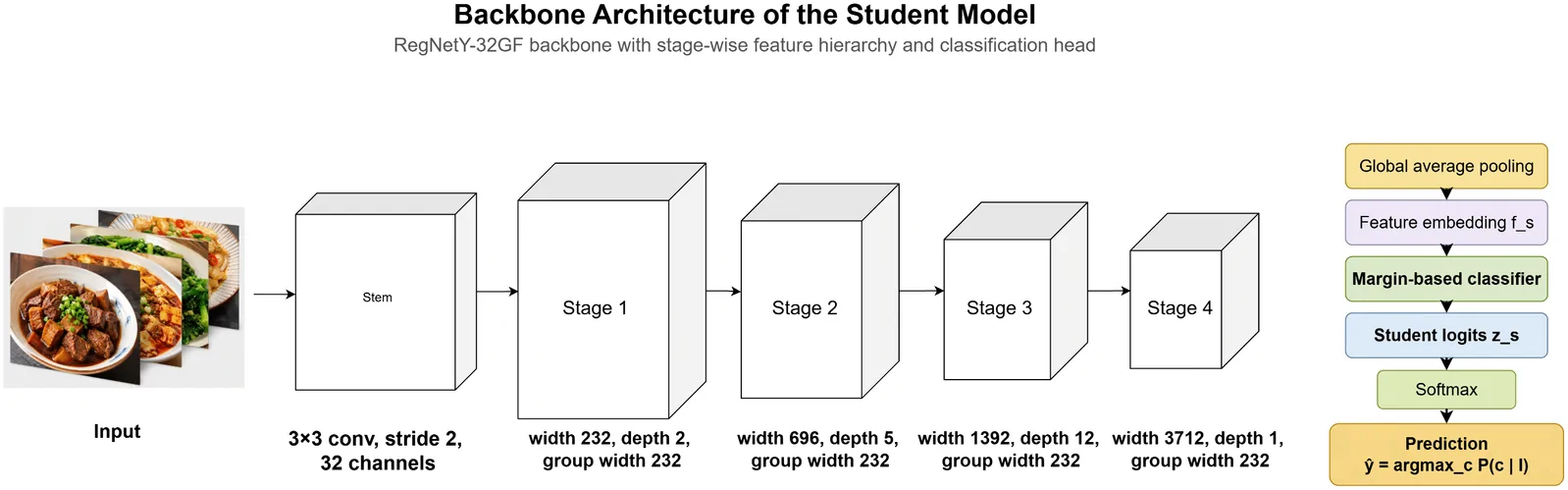
Coordinated discriminative training design of the proposed method. The schematic layout was created by the authors in draw.io, and any food photographs shown as visual examples were taken by the authors.

We constructed an integrated discriminative training strategy around three complementary mechanisms--teacher-guided knowledge distillation, margin-based classification, and embedding regularization--and combined them with mixed-sample augmentation to improve robustness. As shown in Fig 4, a baseline model was first trained and then frozen as the teacher network. During student training, the teacher and student received the same input image and generated teacher and student outputs, respectively [5,16,22,23]. The revised manuscript describes the component interactions as context-dependent complementarity rather than assuming uniform synergy a priori.

Unlike the baseline model, which relied on standard end-to-end supervision, the proposed design aimed to improve category-level separability through teacher-guided knowledge distillation, margin-based classification, triplet-based embedding regularization, and mixed-sample augmentation. Teacher-guided knowledge distillation transferred similarity-aware soft targets from a frozen teacher model. Margin-based classification enlarged angular separation at the classifier level. Triplet-based embedding regularization encouraged tighter intra-class clustering and clearer inter-class separation in feature space. Mixed-sample augmentation was used as a robustness-enhancing regularizer rather than as an independent discriminative objective.

The design principle was not simple module accumulation. Knowledge distillation stabilized the student’s output structure by exposing inter-class similarity patterns from the teacher. Margin-based classification sharpened the class boundary in angular space, whereas embedding regularization improved local metric structure by pulling same-class samples closer and pushing confusing negatives farther apart. Mixed-sample augmentation regularized the model against overconfident fitting to limited presentation patterns. Because the component effects were not assumed to be uniformly additive, pairwise and multi-component diagnostic ablation experiments were added to examine their context-dependent interactions.

Knowledge distillation was implemented through a frozen teacher network. Let ${\text{z}}_{\text{i}}^{\text{t}}$ and ${\text{z}}_{\text{i}}^{\text{s}}$ denote the teacher and student logits for class i, respectively. After temperature scaling, the softened class distributions were defined as [16,22,23]:


$$\begin{array}{c} q_{i}^{\left(T\right)}=\text{exp}\left(z_{i}^{t}/T\right)/\Sigma_{\text{j}}\text{exp}\left(z_{j}^{t}/T\right) \end{array}$$ (2)

$$\begin{array}{c} p_{i}^{\left(T\right)}=\text{exp}\left(z_{i}^{s}/T\right)/\Sigma_{\text{j}}\text{exp}\left(z_{i}^{s}/T\right) \end{array}$$ (3)

$$\begin{array}{c} {\text{L}}_{\text{KD}}\;=\;{\text{T}}^{\text{2}}\;\cdot \;\text{KL}\left(q^{\left(T\right)}\;|\;p^{\left(T\right)}\right) \end{array}$$ (4)

Here, T is the temperature parameter and KL(·||·) denotes the Kullback–Leibler divergence. This formulation encourages the student to match the teacher’s softened output distribution rather than relying only on one-hot labels. In fine-grained recognition, such soft targets carry informative inter-class similarity structure—often referred to as dark knowledge—which is useful when multiple dish categories are visually close and easily confused [16,22,23].

To increase inter-class separation, the conventional classifier was replaced by a margin-based classifier implemented with an ArcFace-style objective [17,24,25]:


$$\begin{array}{c} {\text{L}}_{\text{cl}s}\;=\;-\text{log}\left(\;{\text{e}}^{\left(\text{s}\;\text{cos}\left(\theta_{\text{y}}\;+\;\text{m}\right)\right)}\;/\;\left(\;{\text{e}}^{\left(\text{s}\;\text{cos}\left(\theta_{\text{y}}\;+\;\text{m}\right)\right)}\;+\;\Sigma_{\left(\text{j}\neq \text{y}\right)}\;{\text{e}}^{\left(\text{s}\;\text{cos}\;\theta_{\text{j}}\right)}\;\right)\;\right) \end{array}$$ (5)

In this formulation, s is the feature scale factor, $\theta_{\text{y}}$ is the target-class angle, and m is the additive angular margin. Compared with conventional softmax supervision, the margin term explicitly enlarges angular class separation on the normalized hypersphere. This is theoretically useful in fine-grained dish recognition because visually similar categories often differ only in subtle local cues and therefore benefit from a stricter angular decision boundary [17,24,25].

To further regularize the embedding space, triplet-loss optimization was introduced as embedding regularization [26–28]:


$$\begin{array}{c} {\text{L}}_{\text{tr}iplrt}\;=\;\text{max}\left(\text{0},\;\text{d}\left({\text{f}}_{\text{a}},\;{\text{f}}_{\text{p}}\right)\;-\;\text{d}\left({\text{f}}_{\text{a}},\;{\text{f}}_{\text{n}}\right)\;+\;{\text{m}}_{\text{t}}\right) \end{array}$$ (6)

Here, ${\text{f}}_{\text{a}},\;{\text{f}}_{\text{p}}$, and $f_{n}$ denote the anchor, positive, and negative embeddings, respectively, d(·,·) denotes the embedding-space distance, and m_t is the triplet margin. The objective enforces the metric-learning constraint $d\left(f_{a},f_{p}\right)+m_{t}<d\left(f_{a},f_{n}\right)$, thereby encouraging tighter intra-class compactness and larger inter-class separation. This complements the classifier-level angular margin by improving the local geometry of the learned feature space [26–28].

Triplet samples were constructed using class-balanced mini-batches with P = 32 classes and K = 4 images per class whenever the batch composition allowed. Within each mini-batch, triplets were selected using in-batch batch-hard mining: for each anchor, the positive sample was the farthest same-class sample in the embedding space and the negative sample was the closest different-class sample. This implementation detail was added because the effectiveness and interpretation of triplet-loss regularization depend strongly on the negative-mining strategy.

The final training objective combined supervised classification, knowledge distillation, and embedding regularization:


$$\begin{array}{c} L=(\text{1}-\alpha )L_{cls}+\alpha T^{\text{2}}L_{KD}+\beta L_{triplet} \end{array}$$ (7)

To improve robustness to presentation variation, mixed-sample augmentation based on MixUp and CutMix was used during training [29,30]. For two training samples, MixUp formed a convex combination of image-label pairs, whereas CutMix replaced a local image patch with a patch from another training example and adjusted the target labels according to the replaced area. The augmentation strategy was used as a robustness-enhancing component and was not interpreted as an independent discriminative objective.

The key hyperparameters in the loss formulation and training procedure, including α, β, T, s, m, and m_t, were fixed before the final repeated-seed evaluation. Their values were selected based on prior literature, preliminary training-stability checks, and standard settings commonly adopted for knowledge distillation, ArcFace-style margin learning, and triplet regularization. No repeated hyperparameter optimization was performed on the evaluation split for the purpose of maximizing the final reported performance. The final hyperparameter values are reported in S1 Table, the corresponding symbol definitions are provided in S2 Table, and the configuration records are available in the project repository to support reproducibility.

### Evaluation metrics

Model performance was evaluated using Top-1 accuracy, Top-5 accuracy, macro F1 score, and weighted F1 score. Because overall accuracy alone is insufficient to characterize behavior on difficult fine-grained categories, we additionally performed category-level analysis using per-class F1 scores, confusion matrices, and Delta F1 values. To assess robustness, the main baseline-versus-full-model comparison was repeated over five random seeds (1, 25, 42, 50, and 100). Statistical validation included per-seed McNemar’s exact tests for paired Top-1 correctness, paired bootstrap confidence intervals for metric differences, and a Wilcoxon signed-rank test for whether the mean class-wise Delta F1 across five seeds was greater than zero. The original seed-42 prediction files were retained for representative visualization and detailed per-class examples, whereas the five-seed mean + /- SD results were treated as the primary robustness summary.

## Results

### Overall performance comparison

Table 1 summarizes the overall quantitative comparison among representative CNFOOD-241 studies, the baseline model, the proposed full model, and additional transformer-family baselines evaluated during revision. To avoid overinterpreting a single training run, the revised manuscript reports the baseline and full-model results primarily as five-seed mean + /- SD values. Across five seeds, the baseline achieved 83.72 + /- 0.30% Top-1 accuracy, 97.64 + /- 0.11% Top-5 accuracy, 83.11 + /- 0.31% macro-F1, and 83.63 + /- 0.31% weighted-F1, whereas the proposed full model achieved 84.37 + /- 0.29% Top-1 accuracy, 97.66 + /- 0.11% Top-5 accuracy, 83.41 + /- 0.57% macro-F1, and 84.27 + /- 0.30% weighted-F1. Paired bootstrap analysis indicated a Top-1 improvement of 0.651 percentage points with a 95% confidence interval of [0.491, 0.817]. The comparison with previously published CNFOOD-241 studies is intended for contextual positioning rather than as a strictly controlled head-to-head evaluation.

**Table 1:** Overall performance comparison with representative CNFOOD-241 studies.

Method	Backbone/ Core design	Top-1 Acc (%)	Top-5 Acc (%)
CNFOOD-241 benchmark [8]	ResNeXt101_32x32d	82.05	97.13
CNFOOD-241 benchmark (fusion) [8]	Stacking-based fusion	82.88	—
ResVMamba [10]	Residual selective state-space model	81.70	96.83
ViT-B/16 baseline (ours, seed 42)	Plain Vision Transformer	82.46	96.67
Swin-B baseline (ours, seed 42)	Hierarchical Vision Transformer	84.35	97.78
Baseline (ours, five-seed mean + /- SD)	RegNetY-32GF	83.72 + /- 0.30	97.64 + /- 0.11
Proposed full model (ours, five-seed mean + /- SD)	RegNetY-32GF + coordinated discriminative training design	84.37 + /- 0.29	97.66 + /- 0.11

Note: Most recent CNFOOD-241 studies follow broadly similar closed-set benchmark settings, but differences in pretraining, augmentation, optimization details, and implementation choices remain. Therefore, this comparison is intended to provide contextual positioning rather than to isolate the effect of any single training component. The values for our baseline and full model are reported as five-seed mean + /- SD values, whereas previously published results are quoted as reported in their respective papers.

### Contribution of discriminative learning mechanisms

To examine the contribution of the proposed training strategy, Table 2 summarizes the ablation results using Top-1 and Top-5 accuracy, which were the metrics consistently available across both the standard single-component ablations and the diagnostic reduced-augmentation combination runs. Among the single components, knowledge distillation produced the most consistent improvement in Top-1 and Top-5 accuracy. MBC and ER improved Top-1 accuracy, but their Top-5 accuracy was lower than that of the baseline. This pattern suggests that margin- and embedding-based constraints can sharpen rank-1 decisions while reducing the probability mass assigned to alternative visually plausible classes in the Top-5 list. The additional diagnostic combination rows further indicate that the component effects are context-dependent rather than strictly additive; therefore, these modules should be interpreted as parts of an integrated training recipe rather than uniformly beneficial additions in isolation. Category-level F1 analyses based on prediction-level outputs are reported separately in Table 3 and the supplementary materials.

**Table 2:** Ablation analysis of individual and diagnostic component combinations on CNFOOD-241.

Training setting	KD	MBC	ER	SA	Top-1(%)	Top-5(%)
Baseline	No	No	No	No	84.04	97.54
Baseline + KD	Yes	No	No	No	84.59	97.85
Baseline + MBC	No	Yes	No	No	84.36	96.81
Baseline + ER	No	No	Yes	No	84.29	96.75
Baseline + SA	No	No	No	Yes	84.56	97.82
Baseline+ KD + MBC*	Yes	Yes	No	No	80.84	95.46
Baseline+ KD + ER*	Yes	No	Yes	No	83.10	97.40
Baseline+ MBC + ER*	No	Yes	Yes	No	81.07	94.21
Baseline+ KD + MBC + ER*	Yes	Yes	Yes	No	80.93	95.72
Proposed full model	Yes	Yes	Yes	Yes	84.84	97.80

Abbreviations: KD, teacher-guided knowledge distillation; MBC, margin-based classification; ER, embedding regularization; SA, mixed-sample augmentation. The results reported in this table correspond to the original seed-42 diagnostic ablation experiments.Rows marked with an asterisk (*) denote diagnostic reduced-augmentation combination experiments used to examine the interaction patterns among discriminative components. These diagnostic results suggest that the effects of individual components were not strictly additive, supporting a more cautious interpretation of the proposed method as an integrated discriminative training design with context-dependent component interactions.

**Table 3:** Summary of class-wise Delta F1 analysis across five random seeds.

Metric	Value
Total number of classes	241
Positive mean Delta F1 classes, n (%)	129 (53.53%)
Negative mean Delta F1 classes, n (%)	111 (46.06%)
Zero mean Delta F1 classes, n (%)	1 (0.41%)
Mean class-wise Delta F1 across five seeds	+0.309 percentage points
Wilcoxon signed-rank test	p = 0.017803
Interpretation	Modest positive class-wise shift

Note: Delta F1 was defined as F1(full model) - F1(baseline). The table reports the five-seed mean per-class Delta F1 analysis. Positive values indicate improved class-wise performance, whereas negative values indicate degraded performance. The corresponding full per-class result files, confusion matrices, and sorted Delta F1 distributions are provided in the project repository and supporting information.

### Category-level performance analysis

Although overall accuracy is widely reported, it does not fully reflect how a model behaves on difficult fine-grained categories. We therefore analyzed class-wise F1 changes across all 241 classes. In the original seed-42 prediction files, the full model increased F1 in 148 categories, decreased F1 in 78 categories, and produced almost no change in 15 categories. As summarized in Table 3, in the revised five-seed mean per-class analysis, 129 categories showed positive mean Delta F1, 111 categories showed negative mean Delta F1, and 1 category was unchanged, with a mean class-wise Delta F1 of 0.309 percentage points. A one-sided Wilcoxon signed-rank test indicated that the mean class-wise Delta F1 was significantly greater than zero (p = 0.017803). These results support a modest positive shift in category-level performance while also showing that the full model did not improve all categories uniformly.


$$\begin{array}{c} F{\text{1}}_{i}=\text{2}\left(P_{i}\times R_{i}\right)/\left(P_{i}+R_{i}\right) \end{array}$$ (8)

Category-wise improvement was measured as:


$$\begin{array}{c} \Delta F{\text{1}}_{i}={F\text{1}}_{i}^{\left(full\right)}-{F\text{1}}_{i}^{\left(base\right)} \end{array}$$ (9)

$$\begin{array}{c} Macro-F\text{1}=\left(\text{1}/C\right)\Sigma_{\left(i=\text{1}\right)}^{C}F{\text{1}}_{i} \end{array}$$ (10)

**Fig 5:**
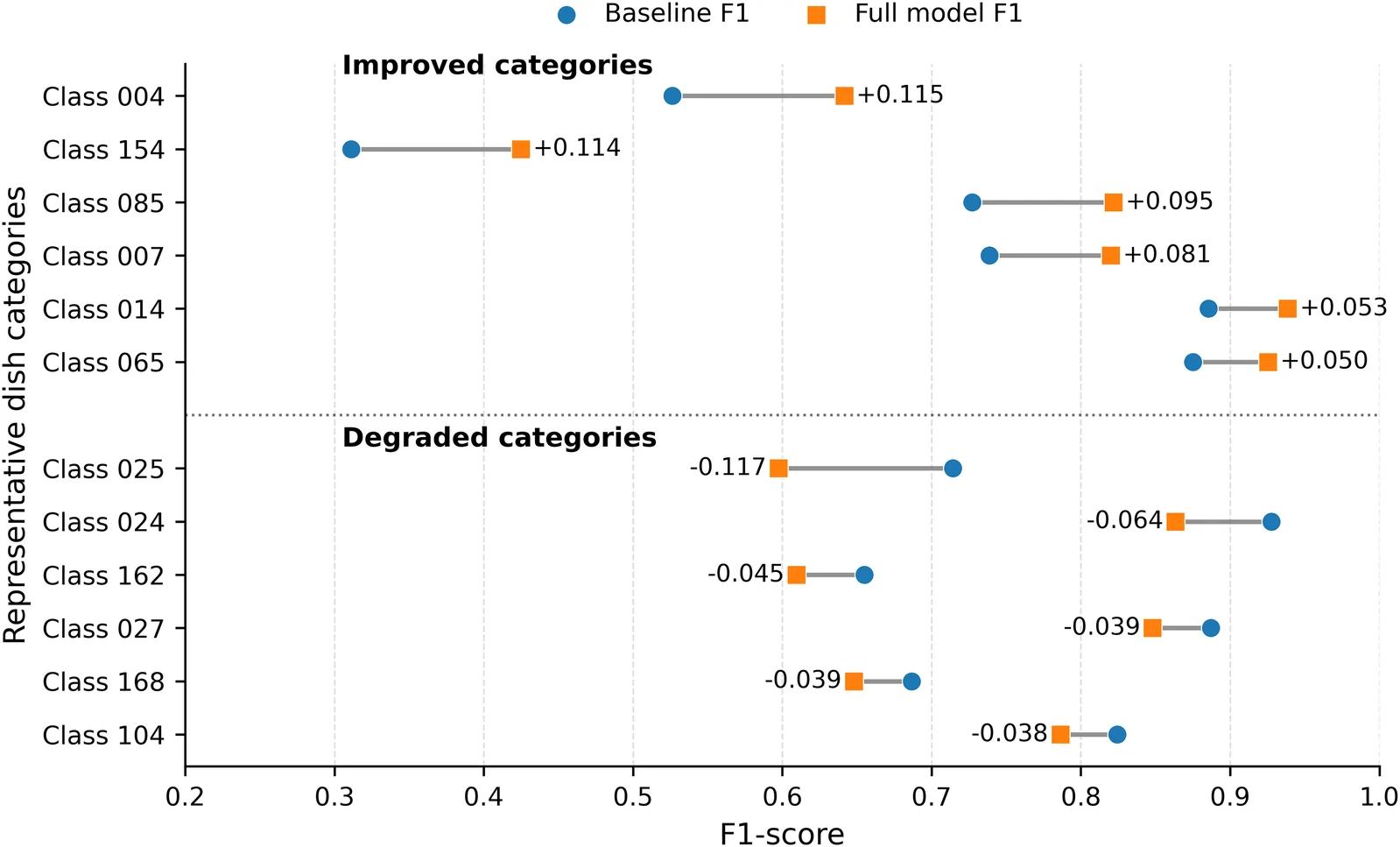
Representative improved and degraded categories based on class-wise F1 changes.

We used F1 score rather than accuracy for category-level analysis because F1 jointly reflects precision and recall and therefore responds directly to changes in false positives and false negatives caused by inter-class confusion [13]. In a fine-grained setting, where the main difficulty lies in separating highly similar subordinate categories [5], reductions in such confusion are expected to appear as improvements in class-wise F1. Macro-F1 gives equal weight to each category and is therefore more informative than overall accuracy when the goal is to determine whether gains are broadly distributed across classes rather than dominated by only a few frequent categories [12,13]. For this reason, the F1-based analysis in this work is interpreted together with confusion matrices, prediction-based hard-category evidence in Fig 2, the balanced improved/degraded visualization in Fig 5, and the full 241-class Delta F1 distribution in S1 Fig.

To avoid selectively presenting only favorable cases, Fig 5 was revised to include both representative improved categories and representative degraded categories. Categories were selected from classes with sufficient validation support (support >= 30), and Delta F1 was defined as F1(full model) - F1(baseline). The revised dumbbell plot shows baseline F1 and full-model F1 for each selected category, allowing the direction and magnitude of the change to be examined simultaneously. This balanced visualization provides a more transparent view of category-level behavior: the full model improves many categories, but some visually ambiguous or unstable categories still degrade, indicating that the proposed training strategy does not solve all fine-grained confusions uniformly.

## Discussion

The present results suggest that improving fine-grained Chinese dish recognition is not simply a matter of using a larger backbone or a more complicated architecture. Instead, the difficulty lies largely in how the model organizes the feature space when confronted with categories that are visually close to one another. The prediction-based hard-category evidence in Fig 2 shows that some low-performing baseline classes are associated with frequent cross-pair confusions, supporting the need for category-level analysis. In this context, a training strategy that explicitly improves representation geometry can be useful, but its effects should be interpreted with the statistical uncertainty and class-specific variability reported in this revision.

The effectiveness of the proposed framework can be understood through the complementary roles of its main components. Teacher-guided knowledge distillation provided similarity-aware soft targets from the teacher model, which conveyed relational information beyond one-hot supervision and helped the student avoid overconfident errors among visually similar dishes [16,22,23]. Margin-based classification strengthened angular separation between classes and made the decision boundary stricter in the embedding space [17,24,25]. Triplet-based embedding regularization further encouraged tighter intra-class clustering and clearer inter-class separation through explicit anchor-positive-negative constraints [26–28]. Mixed-sample augmentation regularized the model against presentation variation and local visual ambiguity [29,30]. However, the diagnostic ablation results indicate that these components are not uniformly additive; their effects are better described as context-dependent complementarity within the complete training recipe.

From a mechanism perspective, knowledge distillation and metric-aware objectives operate at different levels. KD acts on the output distribution and preserves class-relation structure learned by the teacher, whereas ArcFace-style classification and triplet regularization directly reshape the geometry of the student embedding space. Their combination is therefore reasonable: the teacher stabilizes similarity-aware supervision at the distribution level, while the metric-aware terms enforce larger class margins and improved local compactness at the feature level. Nevertheless, because the teacher and student share the same RegNetY-32GF architecture in this self-distillation-like design, the amount of transferable information is constrained by the capacity and biases of the same backbone family. This limitation is now acknowledged explicitly.

An important implication of the current results is that overall accuracy alone is insufficient for evaluating practical progress on CNFOOD-241. In large-scale fine-grained classification, global performance can improve while difficult classes remain unstable. This is why the class-wise analysis shown in Fig 5, together with the complete Delta F1 distribution in S1 Fig, the five-seed class-wise statistics in Table 3, and the prediction-based confusion evidence in Fig 2, is important. These analyses indicate a modest positive shift in category-level performance, but they also show that the proposed method does not uniformly improve all 241 categories.

The prediction-based evidence in Fig 2 further clarifies the importance of a category-level perspective without reproducing original dataset photographs. The selected hard-category patterns show that baseline errors are concentrated in subsets of categories with frequent mutual confusion. Such cases are difficult not only for a standard convolutional baseline, but also for several recent CNFOOD-241 methods that have emphasized the fine-grained and visually confusing nature of the benchmark [8–10]. Therefore, the gains observed in this study should be interpreted in the context of a challenging fine-grained benchmark rather than as routine improvement on a simple closed-set task.

Compared with recent CNFOOD-241 studies, the proposed framework occupies a complementary methodological position. The original CNFOOD-241 work primarily established the benchmark and explored strong CNN backbones and fusion strategies [8]. Subsequent work extended the dataset toward joint classification and nutrient estimation [9], whereas ResVMamba introduced residual selective state-space modeling [10]. In contrast, the present study focused less on redesigning the backbone and more on improving the training objective and feature-space organization within a standard CNN pipeline. Because implementation details, pretraining, and augmentation protocols can differ across studies, the comparison with prior work should be interpreted as contextual positioning rather than a strictly controlled head-to-head evaluation.

Another notable point is the balance between performance and deployability. Because only the trained student branch was retained at inference time, the proposed method preserved a relatively simple inference pipeline even though the training process was more complex. This characteristic may be useful for applications such as intelligent food logging or cloud-assisted dietary analysis, but deployment-oriented claims require additional validation under real-world acquisition conditions.

The additional comparison with the Swin-B backbone provides further context for interpreting the present findings. Although the Transformer-based baseline achieved performance comparable to that of the proposed CNN-based framework, this result should not be interpreted as demonstrating superiority of the proposed method over modern Transformer architectures. Instead, the present study suggests that coordinated discriminative training can improve category-level discrimination within a conventional convolutional backbone without modifying the underlying network architecture. Consequently, the proposed training framework should be regarded as complementary to backbone innovation rather than as an alternative to stronger architectures. Future work may investigate whether the same coordinated discriminative training strategy can further improve Transformer-based backbones.

This study has several limitations. First, even with metric-aware optimization, some highly similar categories remained difficult to separate, and the full model degraded a non-negligible subset of categories in both the original single-run and five-seed analyses. Second, the effectiveness of triplet-loss regularization depends on the quality of positive and negative sample selection, the batch composition, and the triplet margin. Third, the teacher and student shared the same RegNetY-32GF architecture, which limits the diversity of knowledge transferred by the self-distillation-like design. Fourth, although the proposed method achieved improved five-seed mean performance on the standardized closed-set benchmark of CNFOOD-241, this setting mainly reflects category-level discrimination under controlled dataset conditions. In real-world applications, dish recognition may be affected by plating variation, occlusion, illumination changes, cross-restaurant style differences, and the coexistence of multiple food items in a single scene. Therefore, the generalizability of the present framework to more open and complex scenarios still requires further validation.

These limitations suggest several directions for future research. First, more explicit region-aware or attention-based mechanisms could be introduced to enhance the modeling of locally discriminative features, thereby improving the separation of highly similar dish categories. Second, visual recognition could be combined with ingredient priors, recipe information, or multimodal supervision to compensate for the limitations of appearance-only modeling. Finally, once the recognition framework has been further strengthened and its robustness has been validated, it could be extended to nutrition-related downstream applications, such as dish-level nutrient estimation and intelligent dietary assessment [31–33].

The diagnostic reduced-augmentation experiments should also be interpreted with appropriate caution. Although these results suggest context-dependent interactions among the proposed training components, an alternative explanation is that the hyperparameter configuration was selected for the complete training framework and may not be fully optimal after removing the strong mixed-sample augmentation component. Consequently, the observed performance changes may reflect both component interactions and differences in hyperparameter suitability. Therefore, these diagnostic experiments should be interpreted as supportive rather than definitive evidence of context-dependent complementarity. Future work may further clarify the relative contributions of hyperparameter suitability and component interactions through dedicated experimental investigation.

Overall, the results suggest that the proposed integrated discriminative training strategy can provide a modest but statistically supported improvement for fine-grained Chinese dish recognition on CNFOOD-241. The improvement is better interpreted as a context-dependent effect of multiple training mechanisms rather than as evidence of uniform synergy across all components or all categories.

## Conclusions

This study presented an integrated training framework for fine-grained closed-set Chinese dish recognition on the CNFOOD-241 benchmark. Built on a RegNetY-32GF student backbone, the method integrated teacher-guided knowledge distillation, ArcFace-style angular margin classification, triplet-loss regularization, and mixed-sample augmentation to improve the discriminative structure of the learned representation.

Across five random seeds, the proposed full model achieved a higher mean Top-1 accuracy and weighted-F1 score than the baseline, and paired statistical analyses provided evidence of a modest positive improvement. Category-level analysis further showed a positive mean class-wise Delta F1 across five seeds, while also revealing that a subset of categories degraded. These findings support a balanced interpretation: the proposed training strategy improves category-level recognition behavior overall, but its benefit is not uniform across all dish categories.

Compared with recent CNFOOD-241 studies that mainly emphasized model fusion, nutrient-oriented extension, or architecture redesign, this work focused on improving training strategy and feature-space organization within a standard CNN pipeline. This makes the framework conceptually simple and potentially deployable, although future work should further evaluate its robustness under more diverse real-world conditions and against a wider set of modern architectures.

In summary, combining teacher-guided knowledge distillation with metric-aware feature learning produced a modest and statistically supported improvement in fine-grained Chinese dish recognition on CNFOOD-241. The publicly released code, evaluation scripts, derived outputs, and trained model weights are intended to support reproducibility and further category-level analysis by future studies.

## Supporting information

S1 Fig Distribution of class-wise Delta F1 across all 241 categories. (PNG)

S1 Table Complete training hyperparameter settings and configuration records for the baseline, ablation, and full-model experiments. (DOCX)

S2 Table Definitions and values of alpha, beta, T, s, m, and m_t used in the loss functions. (DOCX)

S1 File Per-image prediction files, per-class metric tables, full confusion matrices, statistical-test outputs, and figure source data. (ZIP)

S2 Fig Training curves, validation curves, and loss trajectories for the revised experiments. (PNG)

S3 Table Five-seed repeated-run results, paired-bootstrap confidence intervals, McNemar’s exact tests, and Wilcoxon signed-rank test results. (DOCX)
