# Supplementary material for: Fine-grained Chinese dish recognition on CNFOOD-241: A discriminative learning approach for improving category-level discrimination
Source: PLoS One. 2026 Sep 25;21(9):e0358248. doi: 10.1371/journal.pone.0358248 (PMC13614605; doi:10.1371/journal.pone.0358248)
Supplement: Supplementary file 1 [file pone.0358248.s008.pdf]

Response to Reviewers

Manuscript ID: PONE-D-26-16982

Title: *Fine-grained Chinese dish recognition on CNFOOD-241: A discriminative learning approach for improving category-level discrimination*

Dear Dr. Rana and Reviewers,

We sincerely thank the Academic Editor and the reviewers for the careful evaluation of our manuscript and for the constructive and detailed comments. We appreciate the time and effort devoted to reviewing our work. The comments were very helpful in identifying important aspects that required clarification and strengthening, particularly regarding statistical validation, reproducibility, data availability, ablation analysis, and the interpretation of the proposed discriminative training strategy.

In response, we have substantially revised the manuscript. Specifically, we added repeated experiments over five random seeds, statistical significance analyses, confidence intervals, pairwise and multi-component ablation experiments, additional error analysis, complete per-class results, and clearer documentation of code and data availability. We also moderated the wording throughout the manuscript to avoid overstatement and to more accurately describe the context-dependent nature of the component interactions.

Because page and line numbers may change during journal formatting, the present response refers to the revised manuscript by sections, tables, figures, and supplementary files. All reviewer comments are reproduced below, followed by our point-by-point responses and a summary of the corresponding revisions.

Response to the Academic Editor and Journal Requirements

Editor Comment 1

Please ensure that the manuscript meets PLOS ONE's style requirements, including file naming.

Response:

Thank you for this instruction. We have revised the manuscript format according to the PLOS ONE style requirements and prepared the submission files using the journal's required labels.

Changes made:

The required files are prepared as separate documents labeled as Manuscript, Revised Manuscript with Track Changes, and Response to Reviewers.

Editor Comment 2

Author-generated code underpinning the findings should be made available without restrictions upon publication.

Response:

We agree and have made the training code, evaluation scripts, statistical analysis scripts, trained model weights, and evaluation outputs publicly available through the project repository:

https://github.com/MrShu86/Fine-grained-Chinese-dish-recognition-on-CNFOOD-241

The trained model weights are hosted through the GitHub Release:

https://github.com/MrShu86/Fine-grained-Chinese-dish-recognition-on-CNFOOD-241/releases/tag/revision-model-weights-v1

Changes made:

We revised the Code Availability statement to provide both the GitHub repository link and the GitHub Release link for model weights. The repository includes not only the source code and evaluation scripts, but also the complete five-seed repeated-experiment outputs, the additional ViT-B/16 and Swin-B baseline outputs, the newly added ablation-experiment outputs, and the corresponding trained model weights hosted through the GitHub Release.

Evidence / Results:

The repository includes the baseline RegNetY-32GF code, the proposed discriminative training code, evaluation scripts, statistical analysis scripts, Transformer baseline code, repeated-run outputs, Transformer baseline outputs, ablation outputs, and the corresponding trained model weights. The trained model weights are hosted through the GitHub Release at: https://github.com/MrShu86/Fine-grained-Chinese-dish-recognition-on-CNFOOD-241/releases/tag/revision-model-weights-v1

Editor Comment 3

The Data Availability Statement is missing the repository name and/or DOI/accession number or a direct link to access each database.

Response:

Thank you for pointing this out. We have revised the Data Availability statement to provide a direct reference to the CNFOOD-241 dataset source and to clarify which data were used in this study.

Changes made:

The Data Availability statement has been updated in the manuscript.

Evidence / Results:

The study uses CNFOOD-241, a publicly available benchmark dataset deposited in Mendeley Data under DOI 10.17632/fspyss5zbb.1. The dataset is distributed under the CC BY-NC 3.0 licence description. The revised Data Availability statement provides the dataset DOI, licence information, and the project repository link for the code, evaluation scripts, model weights, and generated evaluation outputs. We do not redistribute the raw CNFOOD-241 images; instead, we provide all derived outputs generated in this study, including prediction

files, per-class metric tables, confusion matrices, statistical outputs, figure source data, and reproducibility scripts through the project repository.

Editor Comment 4

Please ensure that the corresponding author ORCID iD is validated in Editorial Manager.

Response:

Thank you for the reminder. The corresponding author will provide and validate the ORCID iD in Editorial Manager before resubmission.

Changes made:

The corresponding author ORCID iD will be added and validated in Editorial Manager during resubmission.

Editor Comment 5

Figures 1-4 contain copyrighted images; provide CC BY 4.0 permission or remove/replace figures.

Response:

Thank you for raising this important point. We revised the figures to remove ambiguity about photographic materials and third-party copyright. Figures 1, 3, and 4 now use food photographs taken by the authors as visual examples rather than photographs from published articles, websites, or the CNFOOD-241 dataset. Figure 2 was also revised to avoid using dataset photographs; it now uses visualization-derived evidence to support the point about dataset difficulty and prediction behavior. Therefore, the revised figures do not include CNFOOD-241 food photographs as figure materials.

Changes made:

The captions of Figs. 1-4 have been revised to clarify that the schematic diagrams and layout elements were created by the authors in draw.io. The captions for Figs. 1, 3, and 4 now state that the food photographs shown as visual examples were taken by the authors. The revised Fig. 2 does not use dataset photographs.

Evidence / Results:

No third-party copyrighted figure panels are reproduced from published articles or websites. In the revised figures, the food photographs used in Figs. 1, 3, and 4 were taken by the authors. The authors own the rights to these photographs and agree to publish them as part of the article under the journal's publication licence. The revised Fig. 2 uses prediction-based visualization evidence rather than dataset photographs, and Fig. 5 presents both improved and degraded category examples. All revised figures are submitted as separate figure files according to the journal's figure submission requirements.

Editor Comment 6

If reviewer comments include recommendations to cite specific previously published works, evaluate whether they are relevant.

Response:

Thank you for the reminder. We carefully reviewed all editor and reviewer comments and found that no specific previously published work was recommended for citation. Accordingly, no additional citation was required in response to this item.

Changes made:

No manuscript change was required for this item.

Response to Reviewer 1

We thank Reviewer 1 for the detailed and technically constructive critique. The comments helped us improve the statistical rigor, reproducibility, ablation design, and interpretation of the manuscript.

Reviewer 1 Comment 1

The loss formulation is mathematically coherent and the benchmark setup is reproducible.

Response:

We thank the reviewer for this positive assessment. We retained the mathematical formulation and further strengthened the reproducibility information by adding implementation details, statistical analysis procedures, and code availability information.

Changes made:

We revised the Methods and Code Availability sections.

Reviewer 1 Comment 2

The core claim of "coordinated" synergy is unsubstantiated because Table 2 only tests isolated components and not pairwise or multi-component combinations.

Response:

We agree with the reviewer. To address this concern, we conducted additional diagnostic reduced-augmentation ablation experiments in which strong augmentation was disabled to isolate the interaction among KD, MBC, and ER. Specifically, we evaluated KD + MBC, KD + ER, MBC + ER, and KD + MBC + ER. Because these diagnostic runs use a reduced-augmentation setting, we do not present them as direct replacements for the full training recipe. They are used to test component interactions. The results show that the component effects are not simply additive. Among the reduced variants, KD + ER was the strongest combination, while variants involving MBC/ArcFace were more sensitive when strong augmentation was removed.

Based on this finding, we revised the manuscript to avoid overstating a uniform "coordinated synergy" claim. We now describe the method as an integrated training recipe with context-dependent complementarity among components.

Changes made:

We added a new diagnostic reduced-augmentation ablation subsection in the Results and revised the Discussion to explain non-additive component interactions. We also softened the wording from "coordinated synergy" to "context-dependent complementarity" where appropriate.

Evidence / Results:

The new pairwise/core ablation results are shown in Response Table 1 and are also provided in results/ablation/ablation_summary.md and artifacts/ablation_logs/.

Response Table 1. Diagnostic reduced-augmentation ablation results.

Variant	Setting	Top-1	Top-5
KD + MBC	KD on, ArcFace/MBC on, ER off, no strong augmentation	80.84%	95.46%
KD + ER	KD on, MBC off, ER on, no strong augmentation	83.10%	97.40%
MBC + ER	KD off, MBC on, ER on, no strong augmentation	81.07%	94.21%
KD + MBC + ER	KD on, MBC on, ER on, no strong augmentation	80.93%	95.72%

Reviewer 1 Comment 3

MBC and ER individually drop Top-5 below baseline, and this anomaly is not explained.

Response:

Thank you for pointing this out. We have added an explanation that the effects of MBC and ER are context-dependent and should not be interpreted as uniformly improving every metric in isolation. In particular, ArcFace-style margin constraints can improve class-boundary separation but may also sharpen predictions in a way that affects Top-5 behavior. The additional pairwise ablations further show that MBC/ArcFace is sensitive to the surrounding training recipe, especially when strong augmentation is disabled.

Changes made:

We revised the Results and Discussion to explicitly discuss the Top-5 anomaly and to state that the reduced variants are diagnostic reduced-augmentation experiments rather than final competitive models.

Evidence / Results:

The diagnostic reduced-augmentation ablation results in Response Table 1 show that KD + ER was more stable than MBC-containing reduced variants, supporting the interpretation that MBC contributes mainly within the complete training context.

Reviewer 1 Comment 4

78/241 categories degrade under the full model; no investigation is provided into which categories worsen or why.

Response:

Thank you for this comment. Our original category-level analysis was intended to show that the overall gain was distributed across a substantial portion of the dataset rather than being driven by only a few frequent categories. In the original single-run analysis, the full model improved the F1 score in 148 categories, decreased it in 78 categories, and produced almost no change in 15 categories. We have retained this interpretation, while revising the text to state more clearly that the improvement is heterogeneous and that a considerable subset of categories remains difficult.

Changes made:

We revised the category-level Results and Discussion to explicitly report both improved and degraded categories, and to explain that the class-wise results should be interpreted together with the full 241-class Delta F1 distribution, confusion matrix, and representative hard-category examples. We also added supporting files containing all per-class F1 scores, degraded categories, and full confusion matrices.

Evidence / Results:

The revised text follows the original manuscript's category-level conclusion: overall accuracy alone is insufficient for evaluating CNFOOD-241, and the class-wise F1 analysis shows broader but non-uniform changes across categories. The added materials include degraded_categories.csv, improved_categories.csv, top_confusions_full.csv, confusion_matrix_baseline.csv, and confusion_matrix_full.csv. The class-level summary is shown in Response Table 2.

Response Table 2. Class-level F1 change summary under two reporting scopes.

Analysis setting	Seed	Improved classes	Degraded classes	Unchanged classes	Mean class Delta F1
Original manuscript single-run prediction files	42	148	78	15	1.038 pp
Repeated-run per-seed analysis	1	122	112	7	0.261 pp
Repeated-run per-seed analysis	25	97	138	6	-0.763 pp
Repeated-run per-seed analysis	42	148	78	15	1.038 pp
Repeated-run per-seed analysis	50	131	103	7	0.363 pp
Repeated-run per-seed analysis	100	110	117	14	0.352 pp
Five-seed mean per-class Delta F1 analysis	1, 25, 42, 50, 100	129	111	1	0.309 pp

Note: the five-seed row was computed by first averaging Delta F1 for each class across the five seeds and then counting the signs of the 241 mean per-class Delta F1 values. Therefore, the numbers in this row are not obtained by averaging the per-seed counts of improved, degraded, and unchanged classes.

Reviewer 1 Comment 5

The nutrient lookup extension is conceptual only and not evaluated; Fig. 1 space should be reduced or moved to future work.

Response:

We agree. The nutrient lookup module was intended only as a conceptual downstream extension and was not experimentally evaluated in this study. We have therefore reduced the visual and textual emphasis of this part in Fig. 1 and revised the manuscript to state explicitly that the present study evaluates closed-set Chinese dish recognition, whereas nutrient lookup is a possible future extension.

Changes made:

We reduced the space assigned to the nutrient lookup component in Fig. 1 and revised the framework description and Discussion to clarify that this component is conceptual only. The nutrient lookup extension is now explicitly described as future work rather than as an evaluated component.

Reviewer 1 Comment 6

All results are single-run point estimates with no repeated experiments or random seed variation reported.

Response:

We agree and have added repeated experiments using five random seeds: 1, 25, 42, 50, and 100. We report the mean and standard deviation across seeds.

Changes made:

We added a repeated-run evaluation subsection and a new repeated-runs table in the Results. In the revised manuscript, the five-seed mean +/- SD values are used as the primary robustness summary, while the original single-seed results are retained only as the run corresponding to the original experimental report.

Evidence / Results:

The five-seed repeated-run results are summarized in Response Table 3 and provided in results/repeated_runs/repeated_runs_table.md and results/repeated_runs/repeated_runs_per_seed_metrics.csv.

Response Table 3. Five-seed repeated-run results by seed and summary row.

Method	Seed / summary	Top-1	Top-5	Macro-F1	Weighted-F1	95% CI for Top-1 mean
Baseline	1	83.71	97.64	83.14	83.66	--
Baseline	25	84.00	97.71	83.60	83.94	--
Baseline	42	84.04	97.45	83.02	83.91	--
Baseline	50	83.48	97.66	82.74	83.37	--
Baseline	100	83.38	97.72	83.01	83.27	--
Baseline	Mean +/- SD	83.72 +/- 0.30	97.64 +/- 0.11	83.11 +/- 0.31	83.63 +/- 0.31	[83.35, 84.09]
Full model	1	84.42	97.53	83.40	84.28	--
Full model	25	84.13	97.73	82.84	84.03	--
Full model	42	84.84	97.81	84.36	84.76	--
Full model	50	84.15	97.64	83.11	84.04	--
Full model	100	84.32	97.59	83.36	84.22	--
Full model	Mean +/- SD	84.37 +/- 0.29	97.66 +/- 0.11	83.41 +/- 0.57	84.27 +/- 0.30	[84.02, 84.73]

Reviewer 1 Comment 7

No significance test is applied; McNemar's test or paired bootstrap is required for the primary baseline vs. full model comparison.

Response:

We agree. We added McNemar's exact test for paired Top-1 correctness and paired bootstrap confidence intervals for the metric improvements. To avoid overstating the independence of repeated seed-image observations, we report McNemar's test for each seed separately as the main paired correctness check and report the pooled five-seed result only as a supplementary sensitivity analysis.

Changes made:

We added a Statistical Analysis subsection in Methods and a new statistical validation table in Results.

Evidence / Results:

Per-seed McNemar's exact tests are provided in results/significance/significance_per_seed.csv and summarized in Response Table 4. Four of the five seeds showed a significant paired Top-1 correctness difference, while one seed was not significant. Because the same validation images are repeated across seeds, the pooled five-seed result is reported only as a supplementary sensitivity analysis rather than as the sole primary test.

Response Table 4. McNemar's exact test for paired Top-1 correctness.

Analysis	Seed	b01	b10	Two-sided p	Interpretation
Per-seed	1	830	680	0.000124	Significant
Per-seed	25	767	739	0.486600	Not significant
Per-seed	42	756	588	5.093e-06	Significant
Per-seed	50	844	705	0.000451	Significant
Per-seed	100	915	718	1.199e-06	Significant
Pooled sensitivity analysis	1, 25, 42, 50, 100	4112	3430	4.266e-15	Supplementary sensitivity analysis

Reviewer 1 Comment 8

No confidence intervals are reported for any metric.

Response:

We agree and now report confidence intervals. We added 95% confidence intervals for repeated-run Top-1 means and paired bootstrap 95% confidence intervals for improvements in Top-1, Top-5, Macro-F1, and Weighted-F1.

Changes made:

We added confidence intervals to the Results tables.

Evidence / Results:

The paired bootstrap 95% CIs for full minus baseline are reported in the statistical validation table, in results/significance/significance_summary.md, and summarized in Response Table 5.

Response Table 5. Paired bootstrap confidence intervals for full model minus baseline.

Metric	Mean improvement	95% CI	Interpretation
Top-1	0.651 pp	[0.491, 0.817]	Positive CI
Top-5	0.022 pp	[-0.047, 0.091]	CI overlaps zero
Macro-F1	0.285 pp	[0.049, 0.540]	Positive CI
Weighted-F1	0.597 pp	[0.436, 0.766]	Positive CI

Reviewer 1 Comment 9

The 241-class Delta F1 distribution is never tested against zero; a Wilcoxon signed-rank test is needed.

Response:

We agree and have added a one-sided Wilcoxon signed-rank test to evaluate whether the class-wise Delta F1 distribution is significantly greater than zero.

Changes made:

We added the Wilcoxon test to the statistical analysis and Results.

Evidence / Results:

The Wilcoxon signed-rank test result is summarized in Response Table 6.

Response Table 6. Wilcoxon signed-rank test for class-wise Delta F1.

Unit of analysis	Hypothesis	Mean class Delta F1	Positive classes	Negative classes	Zero classes	One-sided p
Mean per-class Delta F1 across five seeds	Delta F1 > 0	0.309 pp	129	111	1	0.017803

Reviewer 1 Comment 10

The hyperparameter selection process is unreported, making it unclear whether tuning was performed on the same split used for evaluation.

Response:

Thank you for this important comment. We revised the Methods to clarify the hyperparameter selection process. The values of alpha, beta, T, s, m, and mt were fixed based on prior literature, preliminary training stability checks, and standard settings commonly used for knowledge distillation, ArcFace-style margin learning, and triplet regularization. We did not perform repeated hyperparameter optimization on the validation/test results to maximize the reported performance.

Changes made:

We added a hyperparameter selection paragraph in Methods and retained the complete values in the supplementary hyperparameter tables.

Reviewer 1 Comment 11

Variance of +/-0.3-0.8% is common across deep learning runs; the reported +0.78% gain is indistinguishable from seed variance without repeated runs.

Response:

We agree and addressed this by performing repeated experiments over five seeds. The full model achieved a higher mean Top-1 accuracy than the baseline, and the paired bootstrap confidence interval for Delta Top-1 remained positive.

Changes made:

We added the repeated-run table and statistical validation results.

Evidence / Results:

The repeated-run and paired-bootstrap results relevant to run-to-run variance are summarized in Response Table 7.

Response Table 7. Robustness summary for the primary Top-1 comparison.

Comparison	Baseline Top-1 mean +/- SD	Full model Top-1 mean +/- SD	Delta Top-1	95% CI for Delta Top-1
Five-seed repeated runs	83.72 +/- 0.30	84.37 +/- 0.29	0.651 pp	[0.491, 0.817]

Reviewer 1 Comment 12

Per-class F1 scores for all 241 categories are not deposited.

Response:

We agree. We provided the full per-class F1 results for all 241 categories as supplementary data.

Changes made:

We added a statement in the manuscript indicating that the full per-class F1 table is provided as supplementary material.

Evidence / Results:

The file results/reviewer_stats/all_241_per_class_f1.csv contains the per-class precision, recall, F1, support, and Delta F1 values.

Reviewer 1 Comment 13

The full confusion matrix is referenced in the methodology but never provided.

Response:

We agree and have added the full confusion matrices for both the baseline and full model as supplementary data.

Changes made:

We updated the Methods and Supplementary Materials descriptions.

Evidence / Results:

The supplementary files include results/reviewer_stats/confusion_matrix_baseline.csv and results/reviewer_stats/confusion_matrix_full.csv.

Reviewer 1 Comment 14

Code and weights are promised as future release but not confirmed available at submission.

Response:

We agree with the reviewer and have revised the Code Availability statement. The project repository is now provided at:

https://github.com/MrShu86/Fine-grained-Chinese-dish-recognition-on-CNFOOD-241

The repository contains the training code, evaluation scripts, statistical analysis scripts, model-related files, repeated-run outputs, Transformer baseline outputs, ablation outputs, and the corresponding trained model weights. The trained model weights are hosted through the GitHub Release at: https://github.com/MrShu86/Fine-grained-Chinese-dish-recognition-on-CNFOOD-241/releases/tag/revision-model-weights-v1

Changes made:

We revised the Code Availability statement to replace future-tense wording with a direct repository link.

Evidence / Results:

The public repository includes baseline code, proposed model code, evaluation scripts, repeated-run scripts, statistical analysis scripts, Transformer baseline scripts, repeated-experiment weights and evaluation results, ViT-B/16 and Swin-B weights and evaluation results, and the newly added ablation-experiment weights and evaluation results.

Reviewer 1 Comment 15

Training curves and loss trajectories for each ablation configuration are absent.

Response:

Thank you for this suggestion. We preserved the training logs for the new ablation configurations and added them as supplementary reproducibility material. These logs record the command line, epoch-level training process, validation metrics, and final best checkpoint for each configuration.

Changes made:

We added a supplementary-material note indicating that the ablation training logs are provided for reproducibility.

Evidence / Results:

The files artifacts/ablation_logs/KD_MBC_train.log, artifacts/ablation_logs/KD_ER_train.log, artifacts/ablation_logs/MBC_ER_train.log, and artifacts/ablation_logs/KD_MBC_ER_core_train.log include the command line, training process, validation metrics, and final best Top-1 results for each configuration.

Reviewer 1 Comment 16

Hyperparameter search records are not provided.

Response:

We have clarified that the study did not rely on an extensive validation-set hyperparameter search. Instead, hyperparameters were fixed based on prior literature, preliminary training stability checks, and standard practice for the corresponding loss terms. This clarification has been added to reduce the risk of perceived optimistic bias.

Changes made:

We added a Methods paragraph describing the hyperparameter selection strategy and clarified that the evaluation split was not used for repeated performance-driven tuning.

Reviewer 1 Comment 17

The comparison in Table 1 against prior CNFOOD-241 methods is confounded by unknown differences in pretraining, augmentation, and implementation.

Response:

We agree. We revised the interpretation of Table 1 to make clear that comparisons with prior CNFOOD-241 studies are contextual rather than strictly controlled head-to-head comparisons. We no longer use Table 1 alone to claim superiority over prior work. Instead, we emphasize the controlled baseline-vs-full comparison within our own experimental setting.

Changes made:

We revised the Results and Discussion text around Table 1 to reduce overstatement and to explicitly mention differences in pretraining, augmentation, input resolution, and implementation details.

Evidence / Results:

The controlled comparison within our study is supported by the five-seed repeated experiments and paired statistical tests. We also added two Transformer-family baselines trained within the current experimental setting using 300 x 300 input resolution: ViT-B/16 and Swin-B.

Reviewer 1 Comment 18

No ViT-family or transformer baseline is included.

Response:

We agree that Transformer-family baselines are important. To address this concern, we added two additional baseline experiments using 300 x 300 input resolution: ViT-B/16 and Swin-B. These models were selected to represent a plain ViT architecture and a hierarchical vision Transformer, respectively. Both models were initialized with ImageNet-pretrained weights and trained on the same CNFOOD-241 split using comparable augmentation and optimization settings.

Changes made:

We added the experimental setup for the Transformer baselines to the Methods and added their results to the comparison table.

Evidence / Results:

The added Transformer baseline results are shown in Response Table 8 and provided in results/transformer_baselines/transformer_baselines_table.md.

Response Table 8. Transformer-family baseline results.

Method	Backbone type	Input size	Seed	Top-1	Top-5	Macro-F1	Weighted-F1
ViT-B/16	Plain Vision Transformer	300	42	82.46%	96.67%	81.69%	82.40%
Swin-B	Hierarchical Vision Transformer	300	42	84.35%	97.78%	83.34%	84.29%

These results provide a direct Transformer-family reference under the revised experimental setting. They also support a more balanced interpretation: the proposed method remains competitive with a strong CNN-based discriminative training recipe, while hierarchical Transformer backbones such as Swin-B are also strong baselines on CNFOOD-241.

Reviewer 1 Comment 19

The triplet mining strategy is never specified.

Response:

We agree and have clarified the triplet mining strategy. The embedding regularization uses batch-hard triplet mining within each mini-batch. Training batches are constructed using a balanced P-K sampler, where P

classes and K images per class are sampled in each mini-batch. Within the mini-batch, the hardest positive and hardest negative are selected for each anchor.

Changes made:

We revised the Methods section describing embedding regularization and triplet sampling.

Evidence / Results:

The implementation uses P=32 and K=4 for batch size 128. The triplet loss is computed using batch-hard positive and negative selection within each P-K mini-batch.

Reviewer 1 Comment 20

Fig. 5 selectively shows only positively improved categories; a balanced visualization including degraded categories would be more transparent.

Response:

We agree. We revised the category-level visualization plan to include both improved and degraded categories. We also added complete per-class F1 and degraded-category tables as supplementary data.

Changes made:

Fig. 5 was revised to include representative degraded categories as well as improved categories. The revised figure is submitted as a separate revised figure file and is accompanied by the source data used to generate it.

Evidence / Results:

The added files include Fig5_double_column_source_data_color_fixed.csv, results/reviewer_stats/improved_categories.csv, results/reviewer_stats/degraded_categories.csv, results/repeated_runs/figures/selected/full_seed_42_class_f1_top50.png, and results/repeated_runs/figures/selected/full_seed_42_class_f1_worst30.png.

Reviewer 1 Comment 21

The self-distillation design uses the same architecture for teacher and student, which is a limitation that is not acknowledged.

Response:

We agree. We added this point to the Limitations section. Although same-architecture self-distillation can provide useful soft-target supervision, it may limit the diversity of teacher signals compared with distillation from a stronger or architecturally different teacher.

Changes made:

We revised the Limitations section to explicitly acknowledge this limitation and to suggest heterogeneous or stronger teacher architectures as future work.

Response to Reviewer 2

We thank Reviewer 2 for the positive assessment of the manuscript's motivation and structure, and for the constructive suggestions regarding statistical validation, public release, triplet sampling, ablation analysis, and language revision.

Reviewer 2 Comment 1

The statistical validation would benefit from multiple experimental runs and appropriate significance testing.

Response:

We agree and have added both repeated experiments and paired statistical tests.

Changes made:

We added a repeated-run table, a statistical analysis subsection, and a statistical validation table.

Evidence / Results:

The repeated-run and statistical validation results are summarized in Response Table 9.

Response Table 9. Statistical validation summary for Reviewer 2 Comment 1.

Analysis	Result	Interpretation
Five-seed Top-1, baseline	83.72 +/- 0.30	Primary robustness summary
Five-seed Top-1, full model	84.37 +/- 0.29	Higher mean Top-1 than baseline
Per-seed McNemar's exact test	Significant in 4 of 5 seeds	Paired correctness improvement is mostly consistent across seeds
Paired bootstrap Delta Top-1	0.651 pp, 95% CI [0.491, 0.817]	Positive CI

Reviewer 2 Comment 2

The code, evaluation scripts, and pretrained model weights should be made publicly accessible.

Response:

We agree. We have revised the Code Availability statement and provided the project repository:

https://github.com/MrShu86/Fine-grained-Chinese-dish-recognition-on-CNFOOD-241

Changes made:

The Code Availability statement now includes the repository link and describes the availability of training code, evaluation scripts, statistical analysis scripts, trained model weights, repeated-run outputs, Transformer baseline outputs, and ablation outputs.

Reviewer 2 Comment 3

Add statistical significance analysis to validate the reported improvements.

Response:

We have added McNemar's exact test, paired bootstrap confidence intervals, and Wilcoxon signed-rank testing for class-wise Delta F1.

Changes made:

We added these analyses to the Methods and Results.

Evidence / Results:

The added significance analyses are summarized in Response Table 10.

Response Table 10. Summary of added statistical significance analyses.

Analysis	Unit of analysis	Statistic / result	Interpretation
McNemar's exact test	Per-seed paired Top-1 correctness	Significant in 4 of 5 seeds; p values: 0.000124, 0.486600, 5.093e-06, 0.000451, 1.199e-06	Paired correctness improvement is mostly consistent across seeds
McNemar's exact test	Pooled seed-image observations	b01=4112, b10=3430, p=4.266e-15	Supplementary sensitivity analysis
Paired bootstrap	Full model minus baseline Top-1	0.651 pp, 95% CI [0.491, 0.817]	Positive CI
Paired bootstrap	Full model minus baseline Macro-F1	0.285 pp, 95% CI [0.049, 0.540]	Positive CI
Paired bootstrap	Full model minus baseline Weighted-F1	0.597 pp, 95% CI [0.436, 0.766]	Positive CI
Wilcoxon signed-rank test	Mean class-wise Delta F1 across five seeds	Mean Delta F1=0.309 pp; p=0.017803	Significant positive class-wise shift

Reviewer 2 Comment 4

Publicly release the code, evaluation scripts, and pretrained weights.

Response:

We agree and have updated the manuscript accordingly. The code and evaluation scripts are available through the GitHub repository listed above. The GitHub Release hosts the trained model weights, including the five-seed repeated-experiment weights, the ViT-B/16 and Swin-B weights, and the newly added ablation-experiment weights, together with their evaluation results. The trained model weights are hosted through the GitHub Release at: https://github.com/MrShu86/Fine-grained-Chinese-dish-recognition-on-CNFOOD-241/releases/tag/revision-model-weights-v1

Changes made:

We revised the Code Availability statement.

Reviewer 2 Comment 5

Clarify the triplet sampling strategy.

Response:

We agree and have clarified the strategy in Methods. The triplet term uses batch-hard mining within balanced P-K mini-batches. Each mini-batch samples P classes and K images per class; the hardest positive and hardest negative are selected within the mini-batch for each anchor.

Changes made:

The Methods section now specifies batch-hard triplet mining and the P-K sampling setting.

Reviewer 2 Comment 6

Strengthen the ablation study and include brief error analysis.

Response:

We agree. We added pairwise and core-combination ablation experiments and a brief error analysis including degraded categories and confusion patterns.

Changes made:

We revised the Results to include pairwise/core ablation results and added a balanced category-level error analysis. We also revised the Discussion to interpret the non-additive interactions among KD, MBC, and ER.

Evidence / Results:

The new diagnostic reduced-augmentation ablation experiments show that KD + ER was the strongest reduced combination under no strong augmentation, while MBC-containing variants were more context-sensitive. Error analysis files include degraded categories, top confusion pairs, and full confusion matrices.

Reviewer 2 Comment 7

Perform minor language revision to improve readability.

Response:

Thank you for this suggestion. We revised the manuscript for clarity, precision, and more cautious interpretation. In particular, we reduced overstatement around "coordinated synergy," clarified the scope of the nutrient lookup extension, and revised the Discussion and Limitations.

Changes made:

Language revisions were made throughout the Abstract, Introduction, Methods, Results, Discussion, and Limitations sections.

Reviewer 2 Comment 8

The figures are not good. Increase the resolution.

Response:

Thank you for this helpful suggestion. We revised the figure preparation to improve visual quality and readability. The schematic figures were re-exported at higher resolution, and the line widths, font sizes, and panel spacing were adjusted to improve clarity in the revised manuscript.

Changes made:

Figures 1-5 were regenerated or re-exported at higher resolution for submission. We also improved figure readability by increasing text size, simplifying figure elements where needed, and revising Fig. 5 to show both improved and degraded categories.

Summary of Major Revisions

1. Added five-seed repeated experiments for the baseline and full model.
2. Added McNemar's exact test, paired bootstrap confidence intervals, and Wilcoxon signed-rank testing.
3. Added pairwise and multi-component ablation experiments.
4. Added balanced category-level error analysis, including degraded categories and confusion matrices.
5. Clarified batch-hard triplet mining with balanced P-K sampling.
6. Revised hyperparameter selection description to state that parameters were fixed based on literature, preliminary experiments, and training stability, rather than repeated validation-set tuning.
7. Revised claims around prior-method comparisons and component synergy to avoid overstatement.
8. Added Code Availability information with the GitHub repository link, GitHub Release link for trained model weights, repeated-run outputs, Transformer baseline outputs, and ablation outputs.
9. Clarified that Figures 1-4 are author-created draw.io figures; food photographs in Figs. 1, 3, and 4 were taken by the authors, the authors own the rights to these photographs and agree to publish them under the journal's publication licence, and Fig. 2 no longer uses dataset photographs.
10. Added limitations regarding same-architecture self-distillation and the conceptual nutrient lookup extension.
11. Added ViT-B/16 and Swin-B Transformer-family baselines using 300 x 300 input resolution.
12. Improved figure resolution and readability for the revised submission.

We again thank the Academic Editor and reviewers for their helpful comments. We believe the revisions have substantially improved the rigor, transparency, and reproducibility of the manuscript.
