# Supplementary material for: Fine-grained Chinese dish recognition on CNFOOD-241: A discriminative learning approach for improving category-level discrimination
Source: PLoS One. 2026 Sep 25;21(9):e0358248. doi: 10.1371/journal.pone.0358248 (PMC13614605; doi:10.1371/journal.pone.0358248)
Supplement: Supplementary file 2 [file pone.0358248.s009.pdf]

Response to Reviewers

Manuscript ID: PONE-D-26-16982

Title: *Fine-grained Chinese dish recognition on CNFOOD-241: A discriminative learning approach for improving category-level discrimination*

Dear Academic Editor and Reviewers,

We sincerely thank the Academic Editor and the reviewers for their careful evaluation of our revised manuscript. We appreciate the positive assessment and the constructive suggestions, which helped us further improve the consistency, interpretation, and presentation of the manuscript. All comments have been carefully addressed, and the corresponding revisions are summarized below.

Response to Reviewer 1

Reviewer 1 Comment 1

There is a discrepancy between the baseline Top-1 accuracy reported in Table 1 and Table 2 (84.04% vs. 84.06%). Please clarify and correct the inconsistency.

Response:

Thank you for identifying this inconsistency. After rechecking the original evaluation records, we confirmed that the value of **84.06%** reported in Table 2 resulted from a transcription error introduced during manuscript revision. The correct Top-1 accuracy for the baseline diagnostic run is **84.04%**, and Table 2 has been corrected accordingly.

We would also like to clarify that Table 1 and Table 2 summarize different experimental settings. Table 1 reports the mean ± SD obtained from five independent random-seed experiments, whereas Table 2 presents the original **seed-42** diagnostic ablation experiments. Consequently, the numerical values reported in the two tables are not expected to be identical even after correcting the transcription error.

To avoid future confusion, we have added a clarifying note in the caption of Table 2 indicating that the ablation results correspond to the original seed-42 diagnostic experiments. This correction does not affect any statistical analyses, interpretations, or conclusions presented in the manuscript.

Changes made:

The baseline Top-1 accuracy in Table 2 has been corrected from 84.06% to 84.04%. In addition, a clarifying note has been added to Table 2 indicating that the reported ablation results correspond to **the original seed-42 diagnostic experiments**, whereas Table 1 summarizes the primary five-seed mean ± SD evaluation.

Evidence / Results:

The corrected value is consistent with the original evaluation outputs (metrics.json) and the prediction results publicly available in the project repository.

Reviewer 1 Comment 2

The manuscript should further discuss why the proposed CNN-based framework remains meaningful given that Swin-B achieves comparable performance.

Response:

We thank the reviewer for this valuable suggestion. We agree that the comparison with Swin-B deserves additional discussion. Accordingly, we have expanded the Discussion section to clarify that the scientific contribution of this study is not to demonstrate superiority over modern Transformer architectures. Instead, our objective is to investigate whether coordinated discriminative training can improve category-level discrimination within a fixed convolutional backbone. The newly added discussion emphasizes that backbone innovation and discriminative training represent complementary research directions rather than competing alternatives, and future work may investigate whether the proposed training strategy can also benefit Transformer-based models.

Changes made:

A new discussion paragraph has been added to the Discussion section immediately following the comparison with the Transformer baselines.

Evidence / Results:

The revised Discussion explicitly states that the proposed framework should be interpreted as a training strategy for improving a fixed CNN backbone rather than as evidence that CNNs outperform Transformer architectures.

Reviewer 1 Comment 3

Please acknowledge that the reduced-augmentation diagnostic ablation results may also reflect hyperparameter suitability rather than solely component interactions.

Response:

We appreciate this insightful comment. We agree that the interpretation of the diagnostic reduced-augmentation experiments should be appropriately qualified. Accordingly, we have revised the Discussion to acknowledge that an alternative explanation is that the hyperparameter configuration was selected for the complete training framework and may not be fully optimal after removing the strong mixed-sample augmentation component. Therefore, the observed performance differences may reflect both context-dependent component interactions and differences in hyperparameter suitability. We now explicitly state that these diagnostic experiments should be interpreted as supportive rather than definitive evidence of component complementarity.

Changes made:

An additional paragraph has been added to the Discussion section to discuss this alternative interpretation and to clarify the limitations of the diagnostic reduced-augmentation experiments.

Evidence / Results:

The revised manuscript now explicitly acknowledges this limitation and presents a more balanced interpretation of the diagnostic ablation results.

Reviewer 1 Comment 4

The Abstract should avoid implying broad improvement across all categories and instead acknowledge that the observed improvements are heterogeneous across categories.

Response:

Thank you for this helpful suggestion. We revised the concluding sentence of the Abstract to better reflect the scope of the experimental findings. The revised wording now emphasizes that the proposed framework provides a modest improvement in category-level discrimination, supported by statistical analyses at the dataset level, while explicitly acknowledging that the magnitude of improvement varies across individual categories. This revision avoids overgeneralization and is consistent with the category-level analyses presented in the Results and Discussion.

Changes made:

The concluding statement of the Abstract has been revised accordingly.

Evidence / Results:

The revised Abstract now states that the proposed framework provides a modest improvement in category-level discrimination, with statistically supported evidence at the dataset level, although the magnitude of improvement varies across individual categories.

Closing Statement

We sincerely thank the reviewer once again for the careful re-evaluation of our manuscript. The additional comments have helped us further improve the consistency, clarity, and interpretation of the revised manuscript. We believe that these revisions strengthen the presentation of our work while preserving the original scientific findings, and we respectfully submit the revised manuscript for your further consideration.
