# Supplementary material for: Fine-grained Chinese dish recognition on CNFOOD-241: A discriminative learning approach for improving category-level discrimination
Source: PLoS One. 2026 Sep 25;21(9):e0358248. doi: 10.1371/journal.pone.0358248 (PMC13614605; doi:10.1371/journal.pone.0358248)
Supplement: Supplementary file 3 [file pone.0358248.s002.docx]

S1 Table. Complete training hyperparameter settings and configuration records

The baseline and full model used the same CNFOOD-241 train/validation split. Repeated runs used seeds 1, 25, 42, 50, and 100 unless otherwise stated.

Setting	Baseline	Full model	Diagnostic ablations
Code directory	RegNetY-32GF/	R_KDAT/	R_KDAT/
Backbone	RegNetY-32GF	RegNetY-32GF	RegNetY-32GF
Input resolution	300 x 300	300 x 300	300 x 300
Number of classes	241	241	241
Training/validation folders	CNFOOD-241 train600x600 / val600x600	Same as baseline	Same as baseline
Batch size	128	128	128
Optimizer	AdamW	AdamW	AdamW
Initial learning rate	5e-4	5e-4	5e-4
Weight decay	1e-4	1e-4	1e-4
Learning-rate schedule	5 warmup epochs + cosine decay	5 warmup epochs + cosine decay	5 warmup epochs + cosine decay
Minimum LR ratio	0.01	0.01	0.01
Evaluation frequency	Every epoch	Every epoch	Every epoch
Validation batches	Full validation set	Full validation set	Full validation set
Mixed precision	bf16	bf16	bf16 unless overridden by launcher
Channels-last memory format	Enabled	Enabled	Enabled unless disabled by launcher
Fused AdamW	Enabled	Enabled	Enabled unless disabled by launcher
DataParallel	Enabled	Enabled	Enabled
Main training epochs	30	40	40
Repeated-run seeds	1, 25, 42, 50, 100	1, 25, 42, 50, 100	Seed 42 for diagnostic ablation summary

Component / hyperparameter	Baseline setting	Full-model setting
Primary classification loss	Cross-entropy	Cross-entropy combined with KD and auxiliary losses
Label smoothing	0.0	0.1
Focal loss	Disabled	Enabled
Focal gamma	2.0 listed in config; inactive when focal loss disabled	2.0
MixUp alpha	0.0	0.2
CutMix alpha	0.0	1.0
Random erasing	Disabled	Enabled
Sampler	Uniform	Balanced P-K sampler
P-K sampler setting	Not used	P = 32, K = 4, batch size = 128
Knowledge distillation	Disabled	Enabled
KD alpha	Not used	0.6
KD temperature T	Not used	3.0
Teacher model/checkpoint	Not used	Frozen baseline RegNetY-32GF teacher checkpoint
Teacher batch-normalization statistics	Not used	Frozen
Margin-based classification / ArcFace	Disabled	Enabled
ArcFace scale s	Not used	30.0
ArcFace margin m	Not used	0.35
Embedding regularization / triplet loss	Disabled	Enabled
Triplet mining	Not used	Batch-hard mining within each P-K mini-batch
Triplet margin m_t	Not used	0.3
Triplet loss weight beta	Not used	0.2
Embedding normalization	Not used	Enabled
MixUp/CutMix final schedule	Not used	Final zero epochs = 3

Diagnostic ablation variant	KD	MBC / ArcFace	ER / Triplet	Strong augmentation	Seed	Top-1 (%)	Top-5 (%)
KD + MBC	On	On	Off	Off	42	80.84	95.46
KD + ER	On	Off	On	Off	42	83.10	97.40
MBC + ER	Off	On	On	Off	42	81.07	94.21
KD + MBC + ER	On	On	On	Off	42	80.93	95.72
