# Supplementary material for: Fine-grained Chinese dish recognition on CNFOOD-241: A discriminative learning approach for improving category-level discrimination
Source: PLoS One. 2026 Sep 25;21(9):e0358248. doi: 10.1371/journal.pone.0358248 (PMC13614605; doi:10.1371/journal.pone.0358248)
Supplement: Supplementary file 4 [file pone.0358248.s003.docx]

S2 Table. Definitions and values of alpha, beta, T, s, m, and m_t used in the loss functions

Symbol	Definition	Value used in full model	Where used
alpha	Weight assigned to the KD term in the combined classification objective	0.6	Knowledge distillation
T	Temperature used to soften teacher and student logits for KL-divergence distillation	3.0	Knowledge distillation
s	Feature/logit scale in the ArcFace-style margin-based classification head	30.0	MBC / ArcFace
m	Additive angular margin in the ArcFace-style classification head	0.35	MBC / ArcFace
m_t	Triplet-margin value used in batch-hard triplet mining	0.3	Embedding regularization
beta	Weight of the triplet embedding-regularization term	0.2	Embedding regularization

The values were fixed before the final repeated-seed evaluation based on prior literature, preliminary training-stability checks, and standard settings for KD, ArcFace-style margin learning, and triplet regularization. They were not repeatedly optimized on validation/test results to maximize reported performance.
