# Supplementary material for: Fine-grained Chinese dish recognition on CNFOOD-241: A discriminative learning approach for improving category-level discrimination
Source: PLoS One. 2026 Sep 25;21(9):e0358248. doi: 10.1371/journal.pone.0358248 (PMC13614605; doi:10.1371/journal.pone.0358248)
Supplement: Supplementary file 5 [file pone.0358248.s006.docx]

S5 Table. Repeated-run and statistical validation results

Values in S5.1 are percentages. Differences and confidence intervals in S5.2 are percentage points, defined as full model minus baseline.

S5.1 Five-seed repeated-run summary: per-seed values

Method	Seed	Top-1 (%)	Top-5 (%)	Macro-F1 (%)	Weighted-F1 (%)
Baseline	1	83.71	97.64	83.14	83.66
Baseline	25	84.00	97.71	83.60	83.94
Baseline	42	84.04	97.45	83.02	83.91
Baseline	50	83.48	97.66	82.74	83.37
Baseline	100	83.38	97.72	83.01	83.27
Full model	1	84.42	97.53	83.40	84.28
Full model	25	84.13	97.73	82.84	84.03
Full model	42	84.84	97.81	84.36	84.76
Full model	50	84.15	97.64	83.11	84.04
Full model	100	84.32	97.59	83.36	84.22

S5.1 Five-seed repeated-run summary: mean and standard deviation

Method	Top-1 mean +/- SD	Top-5 mean +/- SD	Macro-F1 mean +/- SD	Top-1 95% CI	Seeds
Baseline	83.72 +/- 0.30	97.64 +/- 0.11	83.11 +/- 0.31	[83.35, 84.09]	1, 25, 42, 50, 100
Full model	84.37 +/- 0.29	97.66 +/- 0.11	83.41 +/- 0.57	[84.02, 84.73]	1, 25, 42, 50, 100

S5.2 Paired bootstrap confidence intervals for full model minus baseline

Metric	Mean difference (pp)	95% CI (pp)	Interpretation
Top-1	0.651	[0.491, 0.817]	CI excludes 0
Top-5	0.022	[-0.047, 0.091]	CI includes 0
Macro-F1	0.285	[0.049, 0.540]	CI excludes 0
Weighted-F1	0.597	[0.436, 0.766]	CI excludes 0

S5.3 Per-seed McNemar's exact tests for paired Top-1 correctness

Seed	b01	b10	Two-sided exact p	Interpretation
1	830	680	1.244e-04	Significant
25	767	739	0.486600	Not significant
42	756	588	5.093e-06	Significant
50	844	705	4.507e-04	Significant
100	915	718	1.199e-06	Significant

b01 denotes baseline wrong/full model correct; b10 denotes baseline correct/full model wrong.

S5.4 Pooled McNemar sensitivity analysis

Comparison	Seed-image pairs	b01 / b10	Discordant pairs	Two-sided exact p
Full model vs Baseline	104,715	4112 / 3430	7542	4.266e-15

S5.5 Wilcoxon signed-rank test for class-wise Delta F1
