# Supplementary material for: Fine-grained Chinese dish recognition on CNFOOD-241: A discriminative learning approach for improving category-level discrimination
Source: PLoS One. 2026 Sep 25;21(9):e0358248. doi: 10.1371/journal.pone.0358248 (PMC13614605; doi:10.1371/journal.pone.0358248)
Supplement: Supplementary file 6 [file pone.0358248.s007.docx]

1. The figures are not good. Increase the resolution.
2. The manuscript addresses an important topic in fine-grained food image recognition and presents a well-structured discriminative learning framework for improving category-level discrimination on the CNFOOD-241 dataset. The methodology is generally sound, and the experimental results suggest that the proposed training strategy contributes to improved performance, particularly in visually similar dish categories.
3. The manuscript is clearly written and organized, and the category-level F1 analysis provides additional insight beyond conventional accuracy metrics. However, several aspects could be further strengthened. In particular, the statistical validation of the reported improvements would benefit from multiple experimental runs and appropriate significance testing to demonstrate the robustness of the findings.
4. Additionally, although the dataset used in this study is publicly available, the code, evaluation scripts, and pretrained model weights should be made publicly accessible in accordance with the journal's data availability requirements.
5. Overall, this study provides a useful contribution to the field of fine-grained food recognition, and the manuscript could be suitable for publication after addressing the above concerns.
