# Supplementary material for: Fine-grained Chinese dish recognition on CNFOOD-241: A discriminative learning approach for improving category-level discrimination
Source: PLoS One. 2026 Sep 25;21(9):e0358248. doi: 10.1371/journal.pone.0358248 (PMC13614605; doi:10.1371/journal.pone.0358248)
Supplement: Peer review history

# Peer review: Decision Letter 0

**DOI:** 10.1371/journal.pone.0358248.r001

**Authors:** Toqir Rana

10 Jun 2026

Dear Dr. Liao,

Thank you for submitting your manuscript to PLOS ONE. After careful consideration, we feel that it has merit but does not fully meet PLOS ONE’s publication criteria as it currently stands. Therefore, we invite you to submit a revised version of the manuscript that addresses the points raised during the review process.

Please submit your revised manuscript by Jul 25 2026 11:59PM. If you will need more time than this to complete your revisions, please reply to this message or contact the journal office at plosone@plos.org. When you're ready to submit your revision, log on to https://www.editorialmanager.com/pone/ and select the 'Submissions Needing Revision' folder to locate your manuscript file.

- A letter that responds to each point raised by the academic editor and reviewer(s). You should upload this letter as a separate file labeled 'Response to Reviewers'.
- A marked-up copy of your manuscript that highlights changes made to the original version. You should upload this as a separate file labeled 'Revised Manuscript with Track Changes'.
- An unmarked version of your revised paper without tracked changes. You should upload this as a separate file labeled 'Manuscript'.

If you would like to make changes to your financial disclosure, please include your updated statement in your cover letter. Guidelines for resubmitting your figure files are available below the reviewer comments at the end of this letter.

If applicable, we recommend that you deposit your laboratory protocols in protocols.io to enhance the reproducibility of your results. Protocols.io assigns your protocol its own identifier (DOI) so that it can be cited independently in the future. For instructions see: https://journals.plos.org/plosone/s/submission-guidelines#loc-laboratory-protocols. Additionally, PLOS ONE offers an option for publishing peer-reviewed Lab Protocol articles, which describe protocols hosted on protocols.io. Read more information on sharing protocols at https://plos.org/protocols?utm_medium=editorial-email&utm_source=authorletters&utm_campaign=protocols.

As the corresponding author, your ORCID iD is verified in the submission system and will appear in the published article. PLOS supports the use of ORCID, and we encourage all coauthors to register for an ORCID iD and use it as well. Please encourage your coauthors to verify their ORCID iD within the submission system before final acceptance, as unverified ORCID iDs will not appear in the published article. *Only*  the individual author can complete the verification step; PLOS staff *cannot*  verify ORCID iDs on behalf of authors.

We look forward to receiving your revised manuscript.

Kind regards,

Toqir Rana, Ph.D.

Academic Editor

PLOS One

Journal Requirements:

When submitting your revision, we need you to address these additional requirements.

1.Please ensure that your manuscript meets PLOS ONE's style requirements, including those for file naming. The PLOS ONE style templates can be found at

https://journals.plos.org/plosone/s/file?id=wjVg/PLOSOne_formatting_sample_main_body.pdf and

https://journals.plos.org/plosone/s/file?id=ba62/PLOSOne_formatting_sample_title_authors_affiliations.pdf

2. Please note that PLOS One has specific guidelines on code sharing for submissions in which author-generated code underpins the findings in the manuscript. In these cases, we expect all author-generated code to be made available without restrictions upon publication of the work. Please review our guidelines at https://journals.plos.org/plosone/s/materials-and-software-sharing#loc-sharing-code and ensure that your code is shared in a way that follows best practice and facilitates reproducibility and reuse.

3. Please note that your Data Availability Statement is currently missing the repository name and/or the DOI/accession number of each dataset OR a direct link to access each database. If your manuscript is accepted for publication, you will be asked to provide these details on a very short timeline. We therefore suggest that you provide this information now, though we will not hold up the peer review process if you are unable.

4. PLOS requires an ORCID iD for the corresponding author in Editorial Manager on papers submitted after December 6th, 2016. Please ensure that you have an ORCID iD and that it is validated in Editorial Manager. To do this, go to ‘Update my Information’ (in the upper left-hand corner of the main menu), and click on the Fetch/Validate link next to the ORCID field. This will take you to the ORCID site and allow you to create a new iD or authenticate a pre-existing iD in Editorial Manager.

5. We note that Figures 1-4 in your submission contain copyrighted images. All PLOS content is published under the Creative Commons Attribution License (CC BY 4.0), which means that the manuscript, images, and Supporting Information files will be freely available online, and any third party is permitted to access, download, copy, distribute, and use these materials in any way, even commercially, with proper attribution. For more information, see our copyright guidelines: http://journals.plos.org/plosone/s/licenses-and-copyright.

We require you to either (1) present written permission from the copyright holder to publish these figures specifically under the CC BY 4.0 license, or (2) remove the figures from your submission:

1. You may seek permission from the original copyright holder of Figures 1-4 to publish the content specifically under the CC BY 4.0 license.

We recommend that you contact the original copyright holder with the Content Permission Form (http://journals.plos.org/plosone/s/file?id=7c09/content-permission-form.pdf) and the following text:

“I request permission for the open-access journal PLOS ONE to publish XXX under the Creative Commons Attribution License (CCAL) CC BY 4.0 (http://creativecommons.org/licenses/by/4.0/). Please be aware that this license allows unrestricted use and distribution, even commercially, by third parties. Please reply and provide explicit written permission to publish XXX under a CC BY license and complete the attached form.”

Please upload the completed Content Permission Form or other proof of granted permissions as an "Other" file with your submission.

In the figure caption of the copyrighted figure, please include the following text: “Reprinted from [ref] under a CC BY license, with permission from [name of publisher], original copyright [original copyright year].”

2. If you are unable to obtain permission from the original copyright holder to publish these figures under the CC BY 4.0 license or if the copyright holder’s requirements are incompatible with the CC BY 4.0 license, please either i) remove the figure or ii) supply a replacement figure that complies with the CC BY 4.0 license. Please check copyright information on all replacement figures and update the figure caption with source information. If applicable, please specify in the figure caption text when a figure is similar but not identical to the original image and is therefore for illustrative purposes only.

6. If the reviewer comments include a recommendation to cite specific previously published works, please review and evaluate these publications to determine whether they are relevant and should be cited. There is no requirement to cite these works unless the editor has indicated otherwise.

Reviewers' comments:

Reviewer's Responses to Questions

**Comments to the Author**

1. Is the manuscript technically sound, and do the data support the conclusions?

Reviewer #1: Partly

Reviewer #2: Partly

**********

2. Has the statistical analysis been performed appropriately and rigorously?

Reviewer #1: No

Reviewer #2: No

**********

3. Have the authors made all data underlying the findings in their manuscript fully available?

The PLOS Data policy

Reviewer #1: No

Reviewer #2: No

**********

4. Is the manuscript presented in an intelligible fashion and written in standard English?

Reviewer #1: Yes

Reviewer #2: Yes

**********

Reviewer #1: Technical soundness (Partly)

The loss formulation (Eq. 2–7) is mathematically coherent and the benchmark setup is reproducible.

Core claim of "coordinated" synergy is unsubstantiated — Table 2 tests only isolated components, never pairwise or multi-component combinations.

MBC and ER individually drop Top-5 below baseline (96.81%, 96.75% vs. 97.54%) — this anomaly is never explained.

78/241 categories (32.4%) degrade under the full model; no investigation is provided into which categories worsen or why.

The nutrient lookup extension is conceptual only and not evaluated — Fig. 1 space should be reduced or moved to future work All results are single-run point estimates with no repeated experiments, no random seed variation reported.

No significance test is applied to any comparison — McNemar's test or paired bootstrap is required for the primary baseline vs. full model comparison.

No confidence intervals reported for any metric.

The 241-class ΔF1 distribution (mean +0.0104, SD 0.0327) is never tested against zero — a Wilcoxon signed-rank test is needed to support the "broader improvement" claim.

Hyperparameter selection process (α, β, T, s, m, m_t) is unreported — it is unclear whether tuning was performed on the same split used for evaluation, risking optimistic bias.

Variance of ±0.3–0.8% is common across deep learning runs; the reported +0.78% gain is indistinguishable from seed variance without repeated runs.Per-class F1 scores for all 241 categories (the data behind Table 3 summary statistics) are not deposited.

Full confusion matrix is referenced in the methodology but never provided.

Code and weights are promised as future release ("will be publicly released") — not confirmed available at submission, violating PLOS policy.

Training curves and loss trajectories for each ablation configuration are absent.

Hyperparameter search records are not provided.The comparison in Table 1 against prior CNFOOD-241 methods is confounded by unknown differences in pretraining, augmentation, and implementation — the footnote acknowledges this but the paper still uses the table to support competitive positioning claims.

No ViT-family or transformer baseline is included despite these being standard in 2025–2026 fine-grained classification literature.

Triplet mining strategy (hard, semi-hard, or random negatives) is never specified — this is a critical implementation detail that affects reproducibility and result interpretation.

Fig. 5 selectively shows only positively improved categories — a balanced visualization including representative degraded categories would be more scientifically transparent.

The self-distillation design (teacher and student share the same architecture) is a known limitation that is not acknowledged as such.

Reviewer #2: The manuscript addresses an important topic in fine-grained food image recognition and presents a well-structured discriminative learning framework for improving category-level discrimination on the CNFOOD-241 dataset. The methodology is generally sound, and the experimental results suggest that the proposed training strategy contributes to improved performance, particularly in visually similar dish categories.

The manuscript is clearly written and organized, and the category-level F1 analysis provides additional insight beyond conventional accuracy metrics. However, several aspects could be further strengthened. In particular, the statistical validation of the reported improvements would benefit from multiple experimental runs and appropriate significance testing to demonstrate the robustness of the findings. Additionally, although the dataset used in this study is publicly available, the code, evaluation scripts, and pretrained model weights should be made publicly accessible in accordance with the journal's data availability requirements.

Overall, this study provides a useful contribution to the field of fine-grained food recognition, and the manuscript could be suitable for publication after addressing the above concerns.

Add statistical significance analysis to validate the reported improvements.

Publicly release the code, evaluation scripts, and pretrained weights.

Clarify the triplet sampling strategy.

Strengthen the ablation study and include brief error analysis.

Perform minor language revision to improve readability.

**********

what does this mean?). If published, this will include your full peer review and any attached files.

If you choose “no”, your identity will remain anonymous but your review may still be made public.

**Do you want your identity to be public for this peer review?**  For information about this choice, including consent withdrawal, please see our Privacy Policy

Reviewer #1: No

Reviewer #2: **Yes:** Dr. Hiba J. Aleqabie

**********

[NOTE: If reviewer comments were submitted as an attachment file, they will be attached to this email and accessible via the submission site. Please log into your account, locate the manuscript record, and check for the action link "View Attachments". If this link does not appear, there are no attachment files.]

To ensure your figures meet our technical requirements, please review our figure guidelines: https://journals.plos.org/plosone/s/figures

You may also use PLOS’s free figure tool, NAAS, to help you prepare publication quality figures: https://journals.plos.org/plosone/s/figures#loc-tools-for-figure-preparation.

NAAS will assess whether your figures meet our technical requirements by comparing each figure against our figure specifications.

Attachment Submitted filename: review.docx

# Author comment: Author response to Decision Letter 1

**DOI:** 10.1371/journal.pone.0358248.r002

8 Jul 2026

Response to Reviewers

Manuscript ID: PONE-D-26-16982

Title: *Fine-grained Chinese dish recognition on CNFOOD-241: A discriminative learning approach for improving category-level discrimination*

Dear Dr. Rana and Reviewers,

We sincerely thank the Academic Editor and the reviewers for the careful evaluation of our manuscript and for the constructive and detailed comments. We appreciate the time and effort devoted to reviewing our work. The comments were very helpful in identifying important aspects that required clarification and strengthening, particularly regarding statistical validation, reproducibility, data availability, ablation analysis, and the interpretation of the proposed discriminative training strategy.

In response, we have substantially revised the manuscript. Specifically, we added repeated experiments over five random seeds, statistical significance analyses, confidence intervals, pairwise and multi-component ablation experiments, additional error analysis, complete per-class results, and clearer documentation of code and data availability. We also moderated the wording throughout the manuscript to avoid overstatement and to more accurately describe the context-dependent nature of the component interactions.

Because page and line numbers may change during journal formatting, the present response refers to the revised manuscript by sections, tables, figures, and supplementary files. All reviewer comments are reproduced below, followed by our point-by-point responses and a summary of the corresponding revisions.

Response to the Academic Editor and Journal Requirements

Editor Comment 1

Please ensure that the manuscript meets PLOS ONE's style requirements, including file naming.

Response:

Thank you for this instruction. We have revised the manuscript format according to the PLOS ONE style requirements and prepared the submission files using the journal's required labels.

Changes made:

The required files are prepared as separate documents labeled as Manuscript, Revised Manuscript with Track Changes, and Response to Reviewers.

Editor Comment 2

Author-generated code underpinning the findings should be made available without restrictions upon publication.

Response:

We agree and have made the training code, evaluation scripts, statistical analysis scripts, trained model weights, and evaluation outputs publicly available through the project repository:

https://github.com/MrShu86/Fine-grained-Chinese-dish-recognition-on-CNFOOD-241

The trained model weights are hosted through the GitHub Release:

https://github.com/MrShu86/Fine-grained-Chinese-dish-recognition-on-CNFOOD-241/releases/tag/revision-model-weights-v1

Changes made:

We revised the Code Availability statement to provide both the GitHub repository link and the GitHub Release link for model weights. The repository includes not only the source code and evaluation scripts, but also the complete five-seed repeated-experiment outputs, the additional ViT-B/16 and Swin-B baseline outputs, the newly added ablation-experiment outputs, and the corresponding trained model weights hosted through the GitHub Release.

Evidence / Results:

The repository includes the baseline RegNetY-32GF code, the proposed discriminative training code, evaluation scripts, statistical analysis scripts, Transformer baseline code, repeated-run outputs, Transformer baseline outputs, ablation outputs, and the corresponding trained model weights. The trained model weights are hosted through the GitHub Release at: https://github.com/MrShu86/Fine-grained-Chinese-dish-recognition-on-CNFOOD-241/releases/tag/revision-model-weights-v1

Editor Comment 3

The Data Availability Statement is missing the repository name and/or DOI/accession number or a direct link to access each database.

Response:

Thank you for pointing this out. We have revised the Data Availability statement to provide a direct reference to the CNFOOD-241 dataset source and to clarify which data were used in this study.

Changes made:

The Data Availability statement has been updated in the manuscript.

Evidence / Results:

The study uses CNFOOD-241, a publicly available benchmark dataset deposited in Mendeley Data under DOI 10.17632/fspyss5zbb.1. The dataset is distributed under the CC BY-NC 3.0 licence description. The revised Data Availability statement provides the dataset DOI, licence information, and the project repository link for the code, evaluation scripts, model weights, and generated evaluation outputs. We do not redistribute the raw CNFOOD-241 images; instead, we provide all derived outputs generated in this study, including prediction files, per-class metric tables, confusion matrices, statistical outputs, figure source data, and reproducibility scripts through the project repository.

Editor Comment 4

Please ensure that the corresponding author ORCID iD is validated in Editorial Manager.

Response:

Thank you for the reminder. The corresponding author will provide and validate the ORCID iD in Editorial Manager before resubmission.

Changes made:

The corresponding author ORCID iD will be added and validated in Editorial Manager during resubmission.

Editor Comment 5

Figures 1-4 contain copyrighted images; provide CC BY 4.0 permission or remove/replace figures.

Response:

Thank you for raising this important point. We revised the figures to remove ambiguity about photographic materials and third-party copyright. Figures 1, 3, and 4 now use food photographs taken by the authors as visual examples rather than photographs from published articles, websites, or the CNFOOD-241 dataset. Figure 2 was also revised to avoid using dataset photographs; it now uses visualization-derived evidence to support the point about dataset difficulty and prediction behavior. Therefore, the revised figures do not include CNFOOD-241 food photographs as figure materials.

Changes made:

The captions of Figs. 1-4 have been revised to clarify that the schematic diagrams and layout elements were created by the authors in draw.io. The captions for Figs. 1, 3, and 4 now state that the food photographs shown as visual examples were taken by the authors. The revised Fig. 2 does not use dataset photographs.

Evidence / Results:

No third-party copyrighted figure panels are reproduced from published articles or websites. In the revised figures, the food photographs used in Figs. 1, 3, and 4 were taken by the authors. The authors own the rights to these photographs and agree to publish them as part of the article under the journal's publication licence. The revised Fig. 2 uses prediction-based visualization evidence rather than dataset photographs, and Fig. 5 presents both improved and degraded category examples. All revised figures are submitted as separate figure files according to the journal's figure submission requirements.

Editor Comment 6

If reviewer comments include recommendations to cite specific previously published works, evaluate whether they are relevant.

Response:

Thank you for the reminder. We carefully reviewed all editor and reviewer comments and found that no specific previously published work was recommended for citation. Accordingly, no additional citation was required in response to this item.

Changes made:

No manuscript change was required for this item.

Response to Reviewer 1

We thank Reviewer 1 for the detailed and technically constructive critique. The comments helped us improve the statistical rigor, reproducibility, ablation design, and interpretation of the manuscript.

Reviewer 1 Comment 1

The loss formulation is mathematically coherent and the benchmark setup is reproducible.

Response:

We thank the reviewer for this positive assessment. We retained the mathematical formulation and further strengthened the reproducibility information by adding implementation details, statistical analysis procedures, and code availability information.

Changes made:

We revised the Methods and Code Availability sections.

Reviewer 1 Comment 2

The core claim of "coordinated" synergy is unsubstantiated because Table 2 only tests isolated components and not pairwise or multi-component combinations.

Response:

We agree with the reviewer. To address this concern, we conducted additional diagnostic reduced-augmentation ablation experiments in which strong augmentation was disabled to isolate the interaction among KD, MBC, and ER. Specifically, we evaluated KD + MBC, KD + ER, MBC + ER, and KD + MBC + ER. Because these diagnostic runs use a reduced-augmentation setting, we do not present them as direct replacements for the full training recipe. They are used to test component interactions. The results show that the component effects are not simply additive. Among the reduced variants, KD + ER was the strongest combination, while variants involving MBC/ArcFace were more sensitive when strong augmentation was removed.

Based on this finding, we revised the manuscript to avoid overstating a uniform "coordinated synergy" claim. We now describe the method as an integrated training recipe with context-dependent complementarity among components.

Changes made:

We added a new diagnostic reduced-augmentation ablation subsection in the Results and revised the Discussion to explain non-additive component interactions. We also softened the wording from "coordinated synergy" to "context-dependent complementarity" where appropriate.

Evidence / Results:

The new pairwise/core ablation results are shown in Response Table 1 and are also provided in results/ablation/ablation_summary.md and artifacts/ablation_logs/.

Response Table 1. Diagnostic reduced-augmentation ablation results.

Variant Setting Top-1 Top-5

KD + MBC KD on, ArcFace/MBC on, ER off, no strong augmentation 80.84% 95.46%

KD + ER KD on, MBC off, ER on, no strong augmentation 83.10% 97.40%

MBC + ER KD off, MBC on, ER on, no strong augmentation 81.07% 94.21%

KD + MBC + ER KD on, MBC on, ER on, no strong augmentation 80.93% 95.72%

Reviewer 1 Comment 3

MBC and ER individually drop Top-5 below baseline, and this anomaly is not explained.

Response:

Thank you for pointing this out. We have added an explanation that the effects of MBC and ER are context-dependent and should not be interpreted as uniformly improving every metric in isolation. In particular, ArcFace-style margin constraints can improve class-boundary separation but may also sharpen predictions in a way that affects Top-5 behavior. The additional pairwise ablations further show that MBC/ArcFace is sensitive to the surrounding training recipe, especially when strong augmentation is disabled.

Changes made:

We revised the Results and Discussion to explicitly discuss the Top-5 anomaly and to state that the reduced variants are diagnostic reduced-augmentation experiments rather than final competitive models.

Evidence / Results:

The diagnostic reduced-augmentation ablation results in Response Table 1 show that KD + ER was more stable than MBC-containing reduced variants, supporting the interpretation that MBC contributes mainly within the complete training context.

Reviewer 1 Comment 4

78/241 categories degrade under the full model; no investigation is provided into which categories worsen or why.

Response:

Thank you for this comment. Our original category-level analysis was intended to show that the overall gain was distributed across a substantial portion of the dataset rather than being driven by only a few frequent categories. In the original single-run analysis, the full model improved the F1 score in 148 categories, decreased it in 78 categories, and produced almost no change in 15 categories. We have retained this interpretation, while revising the text to state more clearly that the improvement is heterogeneous and that a considerable subset of categories remains difficult.

Changes made:

We revised the category-level Results and Discussion to explicitly report both improved and degraded categories, and to explain that the class-wise results should be interpreted together with the full 241-class Delta F1 distribution, confusion matrix, and representative hard-category examples. We also added supporting files containing all per-class F1 scores, degraded categories, and full confusion matrices.

Evidence / Results:

The revised text follows the original manuscript's category-level conclusion: overall accuracy alone is insufficient for evaluating CNFOOD-241, and the class-wise F1 analysis shows broader but non-uniform changes across categories. The added materials include degraded_categories.csv, improved_categories.csv, top_confusions_full.csv, confusion_matrix_baseline.csv, and confusion_matrix_full.csv. The class-level summary is shown in Response Table 2.

Response Table 2. Class-level F1 change summary under two reporting scopes.

Analysis setting Seed Improved classes Degraded classes Unchanged classes Mean class Delta F1

Original manuscript single-run prediction files 42 148 78 15 1.038 pp

Repeated-run per-seed analysis 1 122 112 7 0.261 pp

Repeated-run per-seed analysis 25 97 138 6 -0.763 pp

Repeated-run per-seed analysis 42 148 78 15 1.038 pp

Repeated-run per-seed analysis 50 131 103 7 0.363 pp

Repeated-run per-seed analysis 100 110 117 14 0.352 pp

Five-seed mean per-class Delta F1 analysis 1, 25, 42, 50, 100 129 111 1 0.309 pp

Note: the five-seed row was computed by first averaging Delta F1 for each class across the five seeds and then counting the signs of the 241 mean per-class Delta F1 values. Therefore, the numbers in this row are not obtained by averaging the per-seed counts of improved, degraded, and unchanged classes.

Reviewer 1 Comment 5

The nutrient lookup extension is conceptual only and not evaluated; Fig. 1 space should be reduced or moved to future work.

Response:

We agree. The nutrient lookup module was intended only as a conceptual downstream extension and was not experimentally evaluated in this study. We have therefore reduced the visual and textual emphasis of this part in Fig. 1 and revised the manuscript to state explicitly that the present study evaluates closed-set Chinese dish recognition, whereas nutrient lookup is a possible future extension.

Changes made:

We reduced the space assigned to the nutrient lookup component in Fig. 1 and revised the framework description and Discussion to clarify that this component is conceptual only. The nutrient lookup extension is now explicitly described as future work rather than as an evaluated component.

Reviewer 1 Comment 6

All results are single-run point estimates with no repeated experiments or random seed variation reported.

Response:

We agree and have added repeated experiments using five random seeds: 1, 25, 42, 50, and 100. We report the mean and standard deviation across seeds.

Changes made:

We added a repeated-run evaluation subsection and a new repeated-runs table in the Results. In the revised manuscript, the five-seed mean +/- SD values are used as the primary robustness summary, while the original single-seed results are retained only as the run corresponding to the original experimental report.

Evidence / Results:

The five-seed repeated-run results are summarized in Response Table 3 and provided in results/repeated_runs/repeated_runs_table.md and results/repeated_runs/repeated_runs_per_seed_metrics.csv.

Response Table 3. Five-seed repeated-run results by seed and summary row.

Method Seed / summary Top-1 Top-5 Macro-F1 Weighted-F1 95% CI for Top-1 mean

Baseline 1 83.71 97.64 83.14 83.66 --

Baseline 25 84.00 97.71 83.60 83.94 --

Baseline 42 84.04 97.45 83.02 83.91 --

Baseline 50 83.48 97.66 82.74 83.37 --

Baseline 100 83.38 97.72 83.01 83.27 --

Baseline Mean +/- SD 83.72 +/- 0.30 97.64 +/- 0.11 83.11 +/- 0.31 83.63 +/- 0.31 [83.35, 84.09]

Full model 1 84.42 97.53 83.40 84.28 --

Full model 25 84.13 97.73 82.84 84.03 --

Full model 42 84.84 97.81 84.36 84.76 --

Full model 50 84.15 97.64 83.11 84.04 --

Full model 100 84.32 97.59 83.36 84.22 --

Full model Mean +/- SD 84.37 +/- 0.29 97.66 +/- 0.11 83.41 +/- 0.57 84.27 +/- 0.30 [84.02, 84.73]

Reviewer 1 Comment 7

No significance test is applied; McNemar's test or paired bootstrap is required for the primary baseline vs. full model comparison.

Response:

We agree. We added McNemar's exact test for paired Top-1 correctness and paired bootstrap confidence intervals for the metric improvements. To avoid overstating the independence of repeated seed-image observations, we report McNemar's test for each seed separately as the main paired correctness check and report the pooled five-seed result only as a supplementary sensitivity analysis.

Changes made:

We added a Statistical Analysis subsection in Methods and a new statistical validation table in Results.

Evidence / Results:

Per-seed McNemar's exact tests are provided in results/significance/significance_per_seed.csv and summarized in Response Table 4. Four of the five seeds showed a significant paired Top-1 correctness difference, while one seed was not significant. Because the same validation images are repeated across seeds, the pooled five-seed result is reported only as a supplementary sensitivity analysis rather than as the sole primary test.

Response Table 4. McNemar's exact test for paired Top-1 correctness.

Analysis Seed b01 b10 Two-sided p Interpretation

Per-seed 1 830 680 0.000124 Significant

Per-seed 25 767 739 0.486600 Not significant

Per-seed 42 756 588 5.093e-06 Significant

Per-seed 50 844 705 0.000451 Significant

Per-seed 100 915 718 1.199e-06 Significant

Pooled sensitivity analysis 1, 25, 42, 50, 100 4112 3430 4.266e-15 Supplementary sensitivity analysis

Reviewer 1 Comment 8

No confidence intervals are reported for any metric.

Response:

We agree and now report confidence intervals. We added 95% confidence intervals for repeated-run Top-1 means and paired bootstrap 95% confidence intervals for improvements in Top-1, Top-5, Macro-F1, and Weighted-F1.

Changes made:

We added confidence intervals to the Results tables.

Evidence / Results:

The paired bootstrap 95% CIs for full minus baseline are reported in the statistical validation table, in results/significance/significance_summary.md, and summarized in Response Table 5.

Response Table 5. Paired bootstrap confidence intervals for full model minus baseline.

Metric Mean improvement 95% CI Interpretation

Top-1 0.651 pp [0.491, 0.817] Positive CI

Top-5 0.022 pp [-0.047, 0.091] CI overlaps zero

Macro-F1 0.285 pp [0.049, 0.540] Positive CI

Weighted-F1 0.597 pp [0.436, 0.766] Positive CI

Reviewer 1 Comment 9

The 241-class Delta F1 distribution is never tested against zero; a Wilcoxon signed-rank test is needed.

Response:

We agree and have added a one-sided Wilcoxon signed-rank test to evaluate whether the class-wise Delta F1 distribution is significantly greater than zero.

Changes made:

We added the Wilcoxon test to the statistical analysis and Results.

Evidence / Results:

The Wilcoxon signed-rank test result is summarized in Response Table 6.

Response Table 6. Wilcoxon signed-rank test for class-wise Delta F1.

Unit of analysis Hypothesis Mean class Delta F1 Positive classes Negative classes Zero classes One-sided p

Mean per-class Delta F1 across five seeds Delta F1 > 0 0.309 pp 129 111 1 0.017803

Reviewer 1 Comment 10

The hyperparameter selection process is unreported, making it unclear whether tuning was performed on the same split used for evaluation.

Response:

Thank you for this important comment. We revised the Methods to clarify the hyperparameter selection process. The values of alpha, beta, T, s, m, and mt were fixed based on prior literature, preliminary training stability checks, and standard settings commonly used for knowledge distillation, ArcFace-style margin learning, and triplet regularization. We did not perform repeated hyperparameter optimization on the validation/test results to maximize the reported performance.

Changes made:

We added a hyperparameter selection paragraph in Methods and retained the complete values in the supplementary hyperparameter tables.

Reviewer 1 Comment 11

Variance of +/-0.3-0.8% is common across deep learning runs; the reported +0.78% gain is indistinguishable from seed variance without repeated runs.

Response:

We agree and addressed this by performing repeated experiments over five seeds. The full model achieved a higher mean Top-1 accuracy than the baseline, and the paired bootstrap confidence interval for Delta Top-1 remained positive.

Changes made:

We added the repeated-run table and statistical validation results.

Evidence / Results:

The repeated-run and paired-bootstrap results relevant to run-to-run variance are summarized in Response Table 7.

Response Table 7. Robustness summary for the primary Top-1 comparison.

Comparison Baseline Top-1 mean +/- SD Full model Top-1 mean +/- SD Delta Top-1 95% CI for Delta Top-1

Five-seed repeated runs 83.72 +/- 0.30 84.37 +/- 0.29 0.651 pp [0.491, 0.817]

Reviewer 1 Comment 12

Per-class F1 scores for all 241 categories are not deposited.

Response:

We agree. We provided the full per-class F1 results for all 241 categories as supplementary data.

Changes made:

We added a statement in the manuscript indicating that the full per-class F1 table is provided as supplementary material.

Evidence / Results:

The file results/reviewer_stats/all_241_per_class_f1.csv contains the per-class precision, recall, F1, support, and Delta F1 values.

Reviewer 1 Comment 13

The full confusion matrix is referenced in the methodology but never provided.

Response:

We agree and have added the full confusion matrices for both the baseline and full model as supplementary data.

Changes made:

We updated the Methods and Supplementary Materials descriptions.

Evidence / Results:

The supplementary files include results/reviewer_stats/confusion_matrix_baseline.csv and results/reviewer_stats/confusion_matrix_full.csv.

Reviewer 1 Comment 14

Code and weights are promised as future release but not confirmed available at submission.

Response:

We agree with the reviewer and have revised the Code Availability statement. The project repository is now provided at:

https://github.com/MrShu86/Fine-grained-Chinese-dish-recognition-on-CNFOOD-241

The repository contains the training code, evaluation scripts, statistical analysis scripts, model-related files, repeated-run outputs, Transformer baseline outputs, ablation outputs, and the corresponding trained model weights. The trained model weights are hosted through the GitHub Release at: https://github.com/MrShu86/Fine-grained-Chinese-dish-recognition-on-CNFOOD-241/releases/tag/revision-model-weights-v1

Changes made:

We revised the Code Availability statement to replace future-tense wording with a direct repository link.

Evidence / Results:

The public repository includes baseline code, proposed model code, evaluation scripts, repeated-run scripts, statistical analysis scripts, Transformer baseline scripts, repeated-experiment weights and evaluation results, ViT-B/16 and Swin-B weights and evaluation results, and the newly added ablation-experiment weights and evaluation results.

Reviewer 1 Comment 15

Training curves and loss trajectories for each ablation configuration are absent.

Response:

Thank you for this suggestion. We preserved the training logs for the new ablation configurations and added them as supplementary reproducibility material. These logs record the command line, epoch-level training process, validation metrics, and final best checkpoint for each configuration.

Changes made:

We added a supplementary-material note indicating that the ablation training logs are provided for reproducibility.

Evidence / Results:

The files artifacts/ablation_logs/KD_MBC_train.log, artifacts/ablation_logs/KD_ER_train.log, artifacts/ablation_logs/MBC_ER_train.log, and artifacts/ablation_logs/KD_MBC_ER_core_train.log include the command line, training process, validation metrics, and final best Top-1 results for each configuration.

Reviewer 1 Comment 16

Hyperparameter search records are not provided.

Response:

We have clarified that the study did not rely on an extensive validation-set hyperparameter search. Instead, hyperparameters were fixed based on prior literature, preliminary training stability checks, and standard practice for the corresponding loss terms. This clarification has been added to reduce the risk of perceived optimistic bias.

Changes made:

We added a Methods paragraph describing the hyperparameter selection strategy and clarified that the evaluation split was not used for repeated performance-driven tuning.

Reviewer 1 Comment 17

The comparison in Table 1 against prior CNFOOD-241 methods is confounded by unknown differences in pretraining, augmentation, and implementation.

Response:

We agree. We revised the interpretation of Table 1 to make clear that comparisons with prior CNFOOD-241 studies are contextual rather than strictly controlled head-to-head comparisons. We no longer use Table 1 alone to claim superiority over prior work. Instead, we emphasize the controlled baseline-vs-full comparison within our own experimental setting.

Changes made:

We revised the Results and Discussion text around Table 1 to reduce overstatement and to explicitly mention differences in pretraining, augmentation, input resolution, and implementation details.

Evidence / Results:

The controlled comparison within our study is supported by the five-seed repeated experiments and paired statistical tests. We also added two Transformer-family baselines trained within the current experimental setting using 300 x 300 input resolution: ViT-B/16 and Swin-B.

Reviewer 1 Comment 18

No ViT-family or transformer baseline is included.

Response:

We agree that Transformer-family baselines are important. To address this concern, we added two additional baseline experiments using 300 x 300 input resolution: ViT-B/16 and Swin-B. These models were selected to represent a plain ViT architecture and a hierarchical vision Transformer, respectively. Both models were initialized with ImageNet-pretrained weights and trained on the same CNFOOD-241 split using comparable augmentation and optimization settings.

Changes made:

We added the experimental setup for the Transformer baselines to the Methods and added their results to the comparison table.

Evidence / Results:

The added Transformer baseline results are shown in Response Table 8 and provided in results/transformer_baselines/transformer_baselines_table.md.

Response Table 8. Transformer-family baseline results.

Method Backbone type Input size Seed Top-1 Top-5 Macro-F1 Weighted-F1

ViT-B/16 Plain Vision Transformer 300 42 82.46% 96.67% 81.69% 82.40%

Swin-B Hierarchical Vision Transformer 300 42 84.35% 97.78% 83.34% 84.29%

These results provide a direct Transformer-family reference under the revised experimental setting. They also support a more balanced interpretation: the proposed method remains competitive with a strong CNN-based discriminative training recipe, while hierarchical Transformer backbones such as Swin-B are also strong baselines on CNFOOD-241.

Reviewer 1 Comment 19

The triplet mining strategy is never specified.

Response:

We agree and have clarified the triplet mining strategy. The embedding regularization uses batch-hard triplet mining within each mini-batch. Training batches are constructed using a balanced P-K sampler, where P classes and K images per class are sampled in each mini-batch. Within the mini-batch, the hardest positive and hardest negative are selected for each anchor.

Changes made:

We revised the Methods section describing embedding regularization and triplet sampling.

Evidence / Results:

The implementation uses P=32 and K=4 for batch size 128. The triplet loss is computed using batch-hard positive and negative selection within each P-K mini-batch.

Reviewer 1 Comment 20

Fig. 5 selectively shows only positively improved categories; a balanced visualization including degraded categories would be more transparent.

Response:

We agree. We revised the category-level visualization plan to include both improved and degraded categories. We also added complete per-class F1 and degraded-category tables as supplementary data.

Changes made:

Fig. 5 was revised to include representative degraded categories as well as improved categories. The revised figure is submitted as a separate revised figure file and is accompanied by the source data used to generate it.

Evidence / Results:

The added files include Fig5_double_column_source_data_color_fixed.csv, results/reviewer_stats/improved_categories.csv, results/reviewer_stats/degraded_categories.csv, results/repeated_runs/figures/selected/full_seed_42_class_f1_top50.png, and results/repeated_runs/figures/selected/full_seed_42_class_f1_worst30.png.

Reviewer 1 Comment 21

The self-distillation design uses the same architecture for teacher and student, which is a limitation that is not acknowledged.

Response:

We agree. We added this point to the Limitations section. Although same-architecture self-distillation can provide useful soft-target supervision, it may limit the diversity of teacher signals compared with distillation from a stronger or architecturally different teacher.

Changes made:

We revised the Limitations section to explicitly acknowledge this limitation and to suggest heterogeneous or stronger teacher architectures as future work.

Response to Reviewer 2

We thank Reviewer 2 for the positive assessment of the manuscript's motivation and structure, and for the constructive suggestions regarding statistical validation, public release, triplet sampling, ablation analysis, and language revision.

Reviewer 2 Comment 1

The statistical validation would benefit from multiple experimental runs and appropriate significance testing.

Response:

We agree and have added both repeated experiments and paired statistical tests.

Changes made:

We added a repeated-run table, a statistical analysis subsection, and a statistical validation table.

Evidence / Results:

The repeated-run and statistical validation results are summarized in Response Table 9.

Response Table 9. Statistical validation summary for Reviewer 2 Comment 1.

Analysis Result Interpretation

Five-seed Top-1, baseline 83.72 +/- 0.30 Primary robustness summary

Five-seed Top-1, full model 84.37 +/- 0.29 Higher mean Top-1 than baseline

Per-seed McNemar's exact test Significant in 4 of 5 seeds Paired correctness improvement is mostly consistent across seeds

Paired bootstrap Delta Top-1 0.651 pp, 95% CI [0.491, 0.817] Positive CI

Reviewer 2 Comment 2

The code, evaluation scripts, and pretrained model weights should be made publicly accessible.

Response:

We agree. We have revised the Code Availability statement and provided the project repository:

https://github.com/MrShu86/Fine-grained-Chinese-dish-recognition-on-CNFOOD-241

Changes made:

The Code Availability statement now includes the repository link and describes the availability of training code, evaluation scripts, statistical analysis scripts, trained model weights, repeated-run outputs, Transformer baseline outputs, and ablation outputs.

Reviewer 2 Comment 3

Add statistical significance analysis to validate the reported improvements.

Response:

We have added McNemar's exact test, paired bootstrap confidence intervals, and Wilcoxon signed-rank testing for class-wise Delta F1.

Changes made:

We added these analyses to the Methods and Results.

Evidence / Results:

The added significance analyses are summarized in Response Table 10.

Response Table 10. Summary of added statistical significance analyses.

Analysis Unit of analysis Statistic / result Interpretation

McNemar's exact test Per-seed paired Top-1 correctness Significant in 4 of 5 seeds; p values: 0.000124, 0.486600, 5.093e-06, 0.000451, 1.199e-06 Paired correctness improvement is mostly consistent across seeds

McNemar's exact test Pooled seed-image observations b01=4112, b10=3430, p=4.266e-15 Supplementary sensitivity analysis

Paired bootstrap Full model minus baseline Top-1 0.651 pp, 95% CI [0.491, 0.817] Positive CI

Paired bootstrap Full model minus baseline Macro-F1 0.285 pp, 95% CI [0.049, 0.540] Positive CI

Paired bootstrap Full model minus baseline Weighted-F1 0.597 pp, 95% CI [0.436, 0.766] Positive CI

Wilcoxon signed-rank test Mean class-wise Delta F1 across five seeds Mean Delta F1=0.309 pp; p=0.017803 Significant positive class-wise shift

Reviewer 2 Comment 4

Publicly release the code, evaluation scripts, and pretrained weights.

Response:

We agree and have updated the manuscript accordingly. The code and evaluation scripts are available through the GitHub repository listed above. The GitHub Release hosts the trained model weights, including the five-seed repeated-experiment weights, the ViT-B/16 and Swin-B weights, and the newly added ablation-experiment weights, together with their evaluation results. The trained model weights are hosted through the GitHub Release at: https://github.com/MrShu86/Fine-grained-Chinese-dish-recognition-on-CNFOOD-241/releases/tag/revision-model-weights-v1

Changes made:

We revised the Code Availability statement.

Reviewer 2 Comment 5

Clarify the triplet sampling strategy.

Response:

We agree and have clarified the strategy in Methods. The triplet term uses batch-hard mining within balanced P-K mini-batches. Each mini-batch samples P classes and K images per class; the hardest positive and hardest negative are selected within the mini-batch for each anchor.

Changes made:

The Methods section now specifies batch-hard triplet mining and the P-K sampling setting.

Reviewer 2 Comment 6

Strengthen the ablation study and include brief error analysis.

Response:

We agree. We added pairwise and core-combination ablation experiments and a brief error analysis including degraded categories and confusion patterns.

Changes made:

We revised the Results to include pairwise/core ablation results and added a balanced category-level error analysis. We also revised the Discussion to interpret the non-additive interactions among KD, MBC, and ER.

Evidence / Results:

The new diagnostic reduced-augmentation ablation experiments show that KD + ER was the strongest reduced combination under no strong augmentation, while MBC-containing variants were more context-sensitive. Error analysis files include degraded categories, top confusion pairs, and full confusion matrices.

Reviewer 2 Comment 7

Perform minor language revision to improve readability.

Response:

Thank you for this suggestion. We revised the manuscript for clarity, precision, and more cautious interpretation. In particular, we reduced overstatement around "coordinated synergy," clarified the scope of the nutrient lookup extension, and revised the Discussion and Limitations.

Changes made:

Language revisions were made throughout the Abstract, Introduction, Methods, Results, Discussion, and Limitations sections.

Reviewer 2 Comment 8

The figures are not good. Increase the resolution.

Response:

Thank you for this helpful suggestion. We revised the figure preparation to improve visual quality and readability. The schematic figures were re-exported at higher resolution, and the line widths, font sizes, and panel spacing were adjusted to improve clarity in the revised manuscript.

Changes made:

Figures 1-5 were regenerated or re-exported at higher resolution for submission. We also improved figure readability by increasing text size, simplifying figure elements where needed, and revising Fig. 5 to show both improved and degraded categories.

Summary of Major Revisions

1. Added five-seed repeated experiments for the baseline and full model.

2. Added McNemar's exact test, paired bootstrap confidence intervals, and Wilcoxon signed-rank testing.

3. Added pairwise and multi-component ablation experiments.

4. Added balanced category-level error analysis, including degraded categories and confusion matrices.

5. Clarified batch-hard triplet mining with balanced P-K sampling.

6. Revised hyperparameter selection description to state that parameters were fixed based on literature, preliminary experiments, and training stability, rather than repeated validation-set tuning.

7. Revised claims around prior-method comparisons and component synergy to avoid overstatement.

8. Added Code Availability information with the GitHub repository link, GitHub Release link for trained model weights, repeated-run outputs, Transformer baseline outputs, and ablation outputs.

9. Clarified that Figures 1-4 are author-created draw.io figures; food photographs in Figs. 1, 3, and 4 were taken by the authors, the authors own the rights to these photographs and agree to publish them under the journal's publication licence, and Fig. 2 no longer uses dataset photographs.

10. Added limitations regarding same-architecture self-distillation and the conceptual nutrient lookup extension.

11. Added ViT-B/16 and Swin-B Transformer-family baselines using 300 x 300 input resolution.

12. Improved figure resolution and readability for the revised submission.

We again thank the Academic Editor and reviewers for their helpful comments. We believe the revisions have substantially improved the rigor, transparency, and reproducibility of the manuscript.

Attachment Submitted filename: Response to Reviewers.pdf

# Peer review: Decision Letter 1

**DOI:** 10.1371/journal.pone.0358248.r003

**Authors:** Toqir Rana

3 Aug 2026

Dear Dr. Liao,

Thank you for submitting your manuscript to PLOS ONE. After careful consideration, we feel that it has merit but does not fully meet PLOS ONE’s publication criteria as it currently stands. Therefore, we invite you to submit a revised version of the manuscript that addresses the points raised during the review process.

Please submit your revised manuscript by Sep 17 2026 11:59PM. If you will need more time than this to complete your revisions, please reply to this message or contact the journal office at plosone@plos.org. When you're ready to submit your revision, log on to https://www.editorialmanager.com/pone/ and select the 'Submissions Needing Revision' folder to locate your manuscript file.

- A letter that responds to each point raised by the academic editor and reviewer(s). You should upload this letter as a separate file labeled 'Response to Reviewers'.
- A marked-up copy of your manuscript that highlights changes made to the original version. You should upload this as a separate file labeled 'Revised Manuscript with Track Changes'.
- An unmarked version of your revised paper without tracked changes. You should upload this as a separate file labeled 'Manuscript'.

If you would like to make changes to your financial disclosure, please include your updated statement in your cover letter. Guidelines for resubmitting your figure files are available below the reviewer comments at the end of this letter.

If applicable, we recommend that you deposit your laboratory protocols in protocols.io to enhance the reproducibility of your results. Protocols.io assigns your protocol its own identifier (DOI) so that it can be cited independently in the future. For instructions see: https://journals.plos.org/plosone/s/submission-guidelines#loc-laboratory-protocols. Additionally, PLOS ONE offers an option for publishing peer-reviewed Lab Protocol articles, which describe protocols hosted on protocols.io. Read more information on sharing protocols at https://plos.org/protocols?utm_medium=editorial-email&utm_source=authorletters&utm_campaign=protocols.

As the corresponding author, your ORCID iD is verified in the submission system and will appear in the published article. PLOS supports the use of ORCID, and we encourage all coauthors to register for an ORCID iD and use it as well. Please encourage your coauthors to verify their ORCID iD within the submission system before final acceptance, as unverified ORCID iDs will not appear in the published article. *Only*  the individual author can complete the verification step; PLOS staff *cannot*  verify ORCID iDs on behalf of authors.

We look forward to receiving your revised manuscript.

Kind regards,

Toqir Rana, Ph.D.

Academic Editor

PLOS One

Journal Requirements:

If the reviewer comments include a recommendation to cite specific previously published works, please review and evaluate these publications to determine whether they are relevant and should be cited. There is no requirement to cite these works unless the editor has indicated otherwise.

Please review your reference list to ensure that it is complete and correct. If you have cited papers that have been retracted, please include the rationale for doing so in the manuscript text, or remove these references and replace them with relevant current references. Any changes to the reference list should be mentioned in the rebuttal letter that accompanies your revised manuscript. If you need to cite a retracted article, indicate the article’s retracted status in the References list and also include a citation and full reference for the retraction notice.

Reviewers' comments:

Reviewer's Responses to Questions

**Comments to the Author**

Reviewer #1: All comments have been addressed

Reviewer #2: All comments have been addressed

**********

2. Is the manuscript technically sound, and do the data support the conclusions?

Reviewer #1: Yes

Reviewer #2: Yes

**********

3. Has the statistical analysis been performed appropriately and rigorously?

Reviewer #1: Yes

Reviewer #2: Yes

**********

4. Have the authors made all data underlying the findings in their manuscript fully available?

The PLOS Data policy

Reviewer #1: Yes

Reviewer #2: Yes

**********

5. Is the manuscript presented in an intelligible fashion and written in standard English?

Reviewer #1: Yes

Reviewer #2: Yes

**********

Reviewer #1: Major strengths of the revision

The authors have addressed the previous round's core methodological gaps in good faith: five-seed repeated experiments, per-seed McNemar's exact tests, paired bootstrap confidence intervals, and a Wilcoxon signed-rank test for class-wise Delta F1 are all now present and appropriately interpreted (Tables 1, 3; Response Tables 3–6). The decision to report both improved and degraded categories in Fig. 5, rather than showing only favorable examples, is good scientific practice and directly resolves a legitimate prior criticism. Code, evaluation scripts, and trained weights are confirmed available (not merely promised) via a public GitHub repository and Release, and the dataset citation (Mendeley DOI) is appropriate. The figure/copyright issue raised by the editor appears properly resolved, with author-taken photographs and non-dataset visualization evidence replacing the original figures.

Issues requiring author response before acceptance

Internal data inconsistency between Table 1 and Table 2. Table 2's ablation "Baseline" row reports 84.06% Top-1 accuracy, but none of the five per-seed baseline values reported in Table 1 / Response Table 3 (83.71, 84.00, 84.04, 83.48, 83.38 for seeds 1, 25, 42, 50, 100) match this figure, nor does it match the five-seed mean (83.72 ± 0.30). Please clarify whether Table 2 was generated from a separate, unlisted run, and if so, state this explicitly and justify why the ablation study uses a different baseline reference point than the main robustness analysis. If this is a transcription error, it should be corrected.

The Swin-B comparison undercuts the paper's central claim and is not adequately discussed. The authors' own added Transformer baseline (Table 1) shows Swin-B achieving 84.35% Top-1 / 97.78% Top-5 with no discriminative training machinery at all — statistically indistinguishable from the proposed full model's five-seed mean of 84.37 ± 0.29% / 97.66 ± 0.11%. This is a genuinely important finding that the authors report transparently, but the Discussion does not confront what it implies: that the entire KD + margin + triplet + augmentation pipeline may deliver little or no benefit over simply choosing a stronger off-the-shelf backbone. I recommend the authors add an explicit paragraph addressing this comparison and tempering the practical framing of their contribution accordingly (e.g., positioning the method as competitive with, rather than superior to, strong Transformer baselines under a fixed CNN backbone constraint, which is a fair contribution but should be stated as such).

"Context-dependent complementarity" may have a simpler explanation. The severe performance collapse in the reduced-augmentation diagnostic ablations (e.g., KD+MBC+ER dropping to 80.93% from an 84%+ baseline; Table 2, Response Table 1) is interpreted as evidence of non-additive interaction among components. However, the hyperparameters (α, β, T, s, m, m_t) were fixed based on literature and stability checks for the full four-component recipe, not re-tuned per ablation configuration. An equally plausible explanation is that these hyperparameters are simply miscalibrated for the reduced-augmentation subsets, rather than reflecting a genuine representational interaction. I recommend the Discussion acknowledge this alternative interpretation rather than presenting "context-dependent complementarity" as the sole explanation.

Category-level improvement is close to marginal and this should be reflected more cautiously in the Abstract. Only 129/241 (53.5%) classes show positive mean Delta F1 across seeds — barely better than even splitting. While the Wilcoxon test is significant (p = 0.018) and the paired bootstrap CI for Top-1 is positive, the Macro-F1 improvement CI [0.049, 0.540] is close to zero. The current Abstract language ("modest but statistically supported improvement") is reasonable but could more clearly signal that the class-level effect is fragile and heterogeneous rather than broadly distribut

Reviewer #2: The authors have addressed the concerns raised during the previous round of review in a comprehensive and satisfactory manner. The revised manuscript demonstrates substantial improvements in statistical validation, reproducibility, methodological clarity, and transparency. The addition of repeated experiments, appropriate statistical analyses, expanded ablation studies, improved error analysis, and enhanced data and code availability has significantly strengthened the manuscript

**********

what does this mean?). If published, this will include your full peer review and any attached files.

If you choose “no”, your identity will remain anonymous but your review may still be made public.

**Do you want your identity to be public for this peer review?**  For information about this choice, including consent withdrawal, please see our Privacy Policy

Reviewer #1: No

Reviewer #2: **Yes:** Hiba Jabbar Aleqabie

**********

[NOTE: If reviewer comments were submitted as an attachment file, they will be attached to this email and accessible via the submission site. Please log into your account, locate the manuscript record, and check for the action link "View Attachments". If this link does not appear, there are no attachment files.]

To ensure your figures meet our technical requirements, please review our figure guidelines: https://journals.plos.org/plosone/s/figures

You may also use PLOS’s free figure tool, NAAS, to help you prepare publication quality figures: https://journals.plos.org/plosone/s/figures#loc-tools-for-figure-preparation.

NAAS will assess whether your figures meet our technical requirements by comparing each figure against our figure specifications.

# Author comment: Author response to Decision Letter 2

**DOI:** 10.1371/journal.pone.0358248.r004

4 Aug 2026

Response to Reviewers

Manuscript ID: PONE-D-26-16982

Title: *Fine-grained Chinese dish recognition on CNFOOD-241: A discriminative learning approach for improving category-level discrimination*

Dear Academic Editor and Reviewers,

We sincerely thank the Academic Editor and the reviewers for their careful evaluation of our revised manuscript. We appreciate the positive assessment and the constructive suggestions, which helped us further improve the consistency, interpretation, and presentation of the manuscript. All comments have been carefully addressed, and the corresponding revisions are summarized below.

Response to Reviewer 1

Reviewer 1 Comment 1

There is a discrepancy between the baseline Top-1 accuracy reported in Table 1 and Table 2 (84.04% vs. 84.06%). Please clarify and correct the inconsistency.

Response:

Thank you for identifying this inconsistency. After rechecking the original evaluation records, we confirmed that the value of 84.06% reported in Table 2 resulted from a transcription error introduced during manuscript revision. The correct Top-1 accuracy for the baseline diagnostic run is 84.04%, and Table 2 has been corrected accordingly.

We would also like to clarify that Table 1 and Table 2 summarize different experimental settings. Table 1 reports the mean ± SD obtained from five independent random-seed experiments, whereas Table 2 presents the original seed-42 diagnostic ablation experiments. Consequently, the numerical values reported in the two tables are not expected to be identical even after correcting the transcription error.

To avoid future confusion, we have added a clarifying note in the caption of Table 2 indicating that the ablation results correspond to the original seed-42 diagnostic experiments. This correction does not affect any statistical analyses, interpretations, or conclusions presented in the manuscript.

Changes made:

The baseline Top-1 accuracy in Table 2 has been corrected from 84.06% to 84.04%. In addition, a clarifying note has been added to Table 2 indicating that the reported ablation results correspond to the original seed-42 diagnostic experiments, whereas Table 1 summarizes the primary five-seed mean ± SD evaluation.

Evidence / Results:

The corrected value is consistent with the original evaluation outputs (metrics.json) and the prediction results publicly available in the project repository.

________________________________________

Reviewer 1 Comment 2

The manuscript should further discuss why the proposed CNN-based framework remains meaningful given that Swin-B achieves comparable performance.

Response:

We thank the reviewer for this valuable suggestion. We agree that the comparison with Swin-B deserves additional discussion. Accordingly, we have expanded the Discussion section to clarify that the scientific contribution of this study is not to demonstrate superiority over modern Transformer architectures. Instead, our objective is to investigate whether coordinated discriminative training can improve category-level discrimination within a fixed convolutional backbone. The newly added discussion emphasizes that backbone innovation and discriminative training represent complementary research directions rather than competing alternatives, and future work may investigate whether the proposed training strategy can also benefit Transformer-based models.

Changes made:

A new discussion paragraph has been added to the Discussion section immediately following the comparison with the Transformer baselines.

Evidence / Results:

The revised Discussion explicitly states that the proposed framework should be interpreted as a training strategy for improving a fixed CNN backbone rather than as evidence that CNNs outperform Transformer architectures.

________________________________________

Reviewer 1 Comment 3

Please acknowledge that the reduced-augmentation diagnostic ablation results may also reflect hyperparameter suitability rather than solely component interactions.

Response:

We appreciate this insightful comment. We agree that the interpretation of the diagnostic reduced-augmentation experiments should be appropriately qualified. Accordingly, we have revised the Discussion to acknowledge that an alternative explanation is that the hyperparameter configuration was selected for the complete training framework and may not be fully optimal after removing the strong mixed-sample augmentation component. Therefore, the observed performance differences may reflect both context-dependent component interactions and differences in hyperparameter suitability. We now explicitly state that these diagnostic experiments should be interpreted as supportive rather than definitive evidence of component complementarity.

Changes made:

An additional paragraph has been added to the Discussion section to discuss this alternative interpretation and to clarify the limitations of the diagnostic reduced-augmentation experiments.

Evidence / Results:

The revised manuscript now explicitly acknowledges this limitation and presents a more balanced interpretation of the diagnostic ablation results.

________________________________________

Reviewer 1 Comment 4

The Abstract should avoid implying broad improvement across all categories and instead acknowledge that the observed improvements are heterogeneous across categories.

Response:

Thank you for this helpful suggestion. We revised the concluding sentence of the Abstract to better reflect the scope of the experimental findings. The revised wording now emphasizes that the proposed framework provides a modest improvement in category-level discrimination, supported by statistical analyses at the dataset level, while explicitly acknowledging that the magnitude of improvement varies across individual categories. This revision avoids overgeneralization and is consistent with the category-level analyses presented in the Results and Discussion.

Changes made:

The concluding statement of the Abstract has been revised accordingly.

Evidence / Results:

The revised Abstract now states that the proposed framework provides a modest improvement in category-level discrimination, with statistically supported evidence at the dataset level, although the magnitude of improvement varies across individual categories.

________________________________________

Closing Statement

We sincerely thank the reviewer once again for the careful re-evaluation of our manuscript. The additional comments have helped us further improve the consistency, clarity, and interpretation of the revised manuscript. We believe that these revisions strengthen the presentation of our work while preserving the original scientific findings, and we respectfully submit the revised manuscript for your further consideration.

Attachment Submitted filename: Response_to_Reviewers_auresp_2.pdf

# Peer review: Decision Letter 2

**DOI:** 10.1371/journal.pone.0358248.r005

**Authors:** Toqir Rana

31 Aug 2026

Fine-grained Chinese dish recognition on CNFOOD-241: A discriminative learning approach for improving category-level discrimination

PONE-D-26-16982R2

Dear Dr. Liao,

We’re pleased to inform you that your manuscript has been judged scientifically suitable for publication and will be formally accepted for publication once it meets all outstanding technical requirements.

Within one week, you’ll receive an e-mail detailing the required amendments. When these have been addressed, you’ll receive a formal acceptance letter and your manuscript will be scheduled for publication.

An invoice will be generated when your article is formally accepted. Please note, if your institution has a publishing partnership with PLOS and your article meets the relevant criteria, all or part of your publication costs will be covered. Please make sure your user information is up-to-date by logging into Editorial Manager at Editorial Manager® and clicking the ‘Update My Information' link at the top of the page. For questions related to billing, please contact billing support.

If your institution or institutions have a press office, please notify them about your upcoming paper to help maximize its impact. If they’ll be preparing press materials, please inform our press team as soon as possible -- no later than 48 hours after receiving the formal acceptance. Your manuscript will remain under strict press embargo until 2 pm Eastern Time on the date of publication. For more information, please contact onepress@plos.org.

Kind regards,

Toqir Rana

Academic Editor

PLOS One

Additional Editor Comments (optional):

Reviewers' comments:

Reviewer's Responses to Questions

**Comments to the Author**

Reviewer #1: All comments have been addressed

**********

2. Is the manuscript technically sound, and do the data support the conclusions?

Reviewer #1: Yes

**********

3. Has the statistical analysis been performed appropriately and rigorously?

Reviewer #1: Yes

**********

4. Have the authors made all data underlying the findings in their manuscript fully available?

The PLOS Data policy

Reviewer #1: (No Response)

**********

5. Is the manuscript presented in an intelligible fashion and written in standard English?

Reviewer #1: (No Response)

**********

Reviewer #1: The revised manuscript and the authors' response to reviewers are well-prepared, scientifically sound, and thoroughly address all previous reviewer concerns. Key Highlights of the Revision:Inconsistency Resolved: The baseline Top-1 accuracy transcription error in Table 2 was corrected to 84.04%, and explicit distinctions between multi-seed evaluations and single-seed diagnostic ablations were clarified. Balanced Claims: The authors appropriately framed the framework's contribution relative to Transformer baselines (like Swin-B) and moderated claims regarding dataset-wide gains to highlight heterogeneous, category-level improvements. Methodological Transparency: Proper caveats were added regarding hyperparameter suitability during diagnostic reduced-augmentation experiments. The manuscript meets the technical and editorial standards for publication.

**********

what does this mean?). If published, this will include your full peer review and any attached files.

If you choose “no”, your identity will remain anonymous but your review may still be made public.

**Do you want your identity to be public for this peer review?**  For information about this choice, including consent withdrawal, please see our Privacy Policy

Reviewer #1: No

**********

# Editor report: Acceptance letter

**DOI:** 10.1371/journal.pone.0358248.r006

**Authors:** Toqir Rana

PONE-D-26-16982R2

PLOS One

Dear Dr. Liao,

I'm pleased to inform you that your manuscript has been deemed suitable for publication in PLOS One. Congratulations! Your manuscript is now being handed over to our production team.

At this stage, our production department will prepare your paper for publication. This includes ensuring the following:

* All references, tables, and figures are properly cited

* All relevant supporting information is included in the manuscript submission,

* There are no issues that prevent the paper from being properly typeset

You will receive further instructions from the production team, including instructions on how to review your proof when it is ready. Please keep in mind that we are working through a large volume of accepted articles, so please give us a few days to review your paper and let you know the next and final steps.

Lastly, if your institution or institutions have a press office, please let them know about your upcoming paper now to help maximize its impact. If they'll be preparing press materials, please inform our press team within the next 48 hours. Your manuscript will remain under strict press embargo until 2 pm Eastern Time on the date of publication. For more information, please contact onepress@plos.org.

You will receive an invoice from PLOS for your publication fee after your manuscript has reached the completed accept phase. If you receive an email requesting payment before acceptance or for any other service, this may be a phishing scheme. Learn how to identify phishing emails and protect your accounts at https://explore.plos.org/phishing.

If we can help with anything else, please email us at customercare@plos.org.

Thank you for submitting your work to PLOS One and supporting open access.

Kind regards,

PLOS One Editorial Office Staff

on behalf of

Dr. Toqir Rana

Academic Editor

PLOS One
